# Human resources for health (and rehabilitation): Six Rehab-Workforce Challenges for the century

**DOI:** 10.1186/s12960-017-0182-7

**Published:** 2017-01-23

**Authors:** Tiago S. Jesus, Michel D. Landry, Gilles Dussault, Inês Fronteira

**Affiliations:** 1 Portuguese Ministry of Education, Aggregation of Schools of Escariz, 4540-320 Escariz, Portugal; 2 Doctor of Physical Therapy Division, Duke University Medical Center, Duke University, Box 104002, 27710 Durham, NC United States of America; 3 Duke Global Health Institute, Duke University, Durham, NC United States of America; 4 Global Health and Tropical Medicine (GHTM) & WHO Collaborating Center on Health Workforce Policy and Planning, Institute of Hygiene and Tropical Medicine-NOVA University of Lisbon (IHMT-UNL), Rua da Junqueira 100, 1349-008 Lisbon, Portugal

**Keywords:** Workforce, Rehabilitation, Health services for persons with disabilities, Global health, Health equity, Human rights

## Abstract

**Background:**

People with disabilities face challenges accessing basic rehabilitation health care. In 2006, the United Nations Convention on the Rights of Persons with Disabilities (CRPD) outlined the global necessity to meet the rehabilitation needs of people with disabilities, but this goal is often challenged by the undersupply and inequitable distribution of rehabilitation workers. While the aggregate study and monitoring of the physical rehabilitation workforce has been mostly ignored by researchers or policy-makers, this paper aims to present the ‘challenges and opportunities’ for guiding further long-term research and policies on developing the relatively neglected, highly heterogeneous physical rehabilitation workforce.

**Methods:**

The challenges were identified through a two-phased investigation. Phase 1: critical review of the rehabilitation workforce literature, organized by the availability, accessibility, acceptability and quality (AAAQ) framework. Phase 2: integrate reviewed data into a SWOT framework to identify the strengths and opportunities to be maximized and the weaknesses and threats to be overcome.

**Results:**

The critical review and SWOT analysis have identified the following global situation: (i) needs-based shortages and lack of access to rehabilitation workers, particularly in lower income countries and in rural/remote areas; (ii) deficiencies in the data sources and monitoring structures; and (iii) few exemplary innovations, of both national and international scope, that may help reduce supply-side shortages in underserved areas.

**Discussion:**

Based on the results, we have prioritized the following ‘Six Rehab-Workforce Challenges’: (1) monitoring supply requirements: accounting for rehabilitation needs and demand; (2) supply data sources: the need for structural improvements; (3) ensuring the study of a whole rehabilitation workforce (i.e. not focused on single professions), including across service levels; (4) staffing underserved locations: the rising of education, attractiveness and tele-service; (5) adapt policy options to different contexts (e.g. rural vs urban), even within a country; and (6) develop international solutions, within an interdependent world.

**Conclusions:**

Concrete examples of feasible local, global and research action toward meeting the Six Rehab-Workforce Challenges are provided. Altogether, these may help advance a policy and research agenda for ensuring that an adequate rehabilitation workforce can meet the current and future rehabilitation health needs.

**Electronic supplementary material:**

The online version of this article (doi:10.1186/s12960-017-0182-7) contains supplementary material, which is available to authorized users.

## Background

There is an estimated one billion people with long-term or residual disabilities around the globe: 15% of the world’s population [1]. The prevalence of disability is expected to grow, due to population ageing and to the so-called epidemic of survival [2], as medical advances are turning life-threatening conditions into disabling ones [1, 3, 4]. Disability is increasingly a public health concern [5, 6], not only by its growing prevalence but also due the health disparities people with disabilities face on a daily basis [1, 7–9].

People with disabilities can experience secondary health conditions resulting from their impairments [10, 11] and disproportionally experience higher violence or abuse [12], unintentional injuries [13] and inequitable access to health promotion activities and general healthcare [1, 7, 14–18]. This leads to increased, preventable risks of chronic conditions, poor health outcomes and even premature death [1, 7, 19–21]. Finally, people with disabilities face barriers to access appropriate physical rehabilitation care [1] which can reduce primary disability and help prevent secondary health conditions [10, 11].

This paper focuses on the state of the physical rehabilitation workforce globally and the challenges people face in accessing physical rehabilitation workers. People with rehabilitation need or demand typically include those with long-term physical, cognitive and/or development impairments contributing to limitations in mobility, self-care, other daily activities and/or restricted social participation. People with temporary physical impairments (e.g. from a broken leg, expecting full recovery after rehabilitation) are also, for a period of time, in need for physical rehabilitation.

Access to needed rehabilitation can be problematic for many reasons. First, in lower income countries, where the vast majority of people with disabilities live [1, 22, 23], rehabilitation providers are unavailable or in very small numbers [1, 24, 25]. Second, existing rehabilitation services and workers concentrate in urban locations and are not accessible to numerous people with disabilities living in rural settings [22, 26, 27]. Third, many people have no access to needed rehabilitation due lack of universal health coverage for even basic rehabilitation [1, 28–30]. Finally, people with disabilities typically have lower employment rates, higher health expenditures and lower mobility. Therefore, the costs of services, lack of transportation or lack of physically accessible sites also are access barriers [1, 29–31].

The study and monitoring of the rehabilitation workforce, and how people with disabilities access them, has been mostly ignored by researchers and policy-makers [1, 24, 32–35]. This negligence is inconsistent with the United Nations Convention on the Rights of Persons with Disabilities (CRPD) [36, 37] and many disability/rehabilitation initiatives [1, 8, 38, 39] recognizing that meeting rehabilitation needs of people with disabilities is an issue of health equity, human rights and social justice.

Universal health coverage, a commitment of Member States of the United Nations and a Sustainable Development Goal frequently seen as an ‘ultimate expression of fairness’ [33, 40], cannot in our view be achieved if it does not include the rehabilitation needs of people with disabilities [23, 36, 37, 41].

The rehabilitation health workforce supply consists of many different configurations of professions. This includes physicians specialized in physical medicine and rehabilitation, physical therapists (PTs), occupational therapists (OTs), speech-language pathologists, prosthetic and orthotic practitioners, and PT/OT assistants, among a wide array of other health workers and family supplying the population’s physical rehabilitation needs. In addition to that heterogeneity in its whole composition, the existence, practices, education and competencies of any of those rehabilitation health workers often vary widely across countries, and even within the same country [24, 25].

This paper aims to identify long-term ‘challenges and opportunities’ for advancing the global study, monitoring and development of the relatively neglected, highly heterogeneous, physical rehabilitation workforce. To do so, we have conducted a two-phased investigation:

- Phase 1: critical review of the rehabilitation workforce literature, focusing on the AAAQ framework: the availability, accessibility, acceptability and quality [33] of the physical rehabilitation workforce.
- Phase 2: integration of reviewed data into a SWOT framework [42] to identify the strengths, weaknesses, opportunities and threats for the global advancement of this health workforce and their ability to meet the world’s rehabilitation needs.

## Methods

### Phase 1

Searches for the relevant literature were conducted in PubMed, covering the period between March 2006 and March 2016, using the following MeSH terms: ‘Manpower’ OR ‘Health Manpower’ AND rehabilitation-related terms, abstracted from previous studies finding physical rehabilitation content in PubMed [43, 44]. Additional file 1 details that search strategy.

Secondary searches (citation-tracking, author-tracking, consulting references lists) were also performed. The World Report on Disability [1] was also consulted, both as informative material and source of references.

Papers were primarily selected, and their content abstracted, if published in English and potentially fitting into any category of the AAAQ framework [33]. Table 1 shows category definitions, also used for data synthesis. Except for letters and manuscripts without abstracts, papers describing any research design were considered for inclusion.

**Table 1:** The AAAQ framework: a sequence of four, critical dimensions for analysing human

Framework dimensions	Operational definition
Availability	The sufficient supply, appropriate stock of health workers, with the relevant competencies and skill mix that corresponds to the health needs of the population
Accessibility	The equitable distribution of health workers in terms of travel time and transport (spatial), opening hours and corresponding workforce attendance (temporal), the infrastructure’s attributes (physical—such as disabled-friendly buildings), referral mechanisms (organizational) and the direct and indirect cost of services, both formal and informal (financial)
Acceptability	The characteristics and ability of the workforce to treat all patients with dignity, create trust and enable or promote demand for services; this may take different forms such as a same-sex provider or a provider who understands and speaks one’s language and whose behaviour is respectful according to age, religion, social, cultural values, etc.
Quality	The competencies, skills, knowledge and behaviour of the health worker as assessed according to professional norms (or other guiding standards) and as perceived by users

Source: Campbell J, Dussault G, Buchan J, Pozo-Martin F, Guerra Arias M, Leone C, Siyam A, Cometto G. *A Universal Truth: No Health Without a Workforce: Third Global Forum on Human Resources for Health Report.* Geneva : Global Health Workforce Alliance and World Health Organization, 2014

Papers finally included in the review were selected, at the synthesis stage, according to the following criteria: more recent (since 2008), specific for the (sub-)topic addressed, and whose content was not synthesized/addressed by any included systematic review. Additional file 2 outlines the papers primarily selected but deleted at the synthesis and the reasons to do so. Additional file 3 presents the data extraction table of the papers finally included. An iterative selection alongside the synthesis is characteristic of reviews covering wide/complex healthcare topics, such as this one [21, 45–47].

### Phase 2

A SWOT analysis [42] was conducted to integrate the literature reviewed. It aimed to identify which strengths and opportunities might be maximized as well as which weaknesses and threats might be minimized, eliminated or overcome, toward advancing the study, monitoring and development of the rehabilitation workforce.

Table 2 shows how general definitions of each SWOT analysis category [42] were translated by the authors into operational definitions guiding this study’s analytical process [48, 49].

**Table 2:** General and operational definitions of the SWOT analysis categories for this study

	General definition	Translation into operational definitions for this study
Strengths	Internal properties of the system or organization under study that represent a competitive advantage for that system or its own development	• Aspects that the rehabilitation workforce literature identifies as successful and might be maximized in those specific contexts • Aspects of the rehabilitation workforce literature that inspire, or identify elements in need for, specific improvement action in identified contexts
Weaknesses	Limitation internal to the system or organization under study that may hamper its progress	• Barriers to the progress of the study, monitoring and development of the rehabilitation workforce • Structural barriers impeding the access of people with disabilities to the rehabilitation health workers they need • Aspects that the rehabilitation literature is unable to identify in sufficient detail to trigger any specific improvement action
Opportunities	Any external environmental factor that may act as a facilitator to the progress of the system or organization under study	• Interventions/innovations that the rehabilitation workforce literature reports as successfully applied into one context (e.g. geography) and that might be potentially transferred to other contexts as well—particularly those with higher need • Any relevant contextual factor that may act as facilitator to the advancement of the rehabilitation workforce
Threats	Any external environmental factor that may act as a barrier to the system or organization under study	• Factors external to the advances assisted in the rehabilitation workforce and its literature that may act as a barrier to the progress in the study, monitoring and development of the rehabilitation workforce

Originally from the management literature [42], SWOT analyses have been used successfully in healthcare studies [48, 49], including in one country, Kuwait, to help drawing recommendations for advancing the physical therapy profession [48]. In this paper, it enables the design of ‘challenges’ for the global advancement of the broader physical rehabilitation workforce.

## Results

### Phase 1: critical review of the rehabilitation workforce literature

#### Availability

The rehabilitation workforce literature commonly reports important limitations in the supply data sources [1, 24–26, 50–53].

##### Shortcomings of the supply data

First, mandatory professional registration/licensing mechanisms for rehabilitation workers are absent in many countries, especially lower income countries [24, 32, 50, 54–57]. While international professional associations of PTs and OTs have been collating supply data from their national member organisations, there is no dedicated data source, no standards for data collection at national level and many countries are not represented [32, 53].

Second, the Global Atlas of the Health Workforce provides no data on a specific category of rehabilitation workers, who are typically aggregated under ‘other health workers’, with unrelated professions such as ambulance workers [51].

All of this is complicated by the lack of uniform international definitions/classifications of who are rehabilitation health workers, and by policies that continue to place the monitoring of rehabilitation workers low on the health agenda, in turn related to how societies often interpret and react to disability [1, 24, 50].

Finally, terminologies used to describe the same profession (physical therapists vs physiotherapists; occupational therapist vs ergo-therapists) vary. More importantly, their competencies, education, credentials and typical practices also vary within and across countries or practice locations, for the same profession [1, 24, 25, 58, 59].

##### Variability in determining supply requirements

Determining rehabilitation workers’ supply requirements is made on the basis of population size [32, 53, 60], other need indicators (population ageing, epidemiological variables) [24–26] or even demand indicators (rehabilitation services use, data on unfilled vacancies) [61, 62].

##### Data on availability

Substantial needs-based shortages of rehabilitation workers are documented and projected in many places around the globe [1, 24, 32, 53, 62, 63]. The scenario is worst in lower income countries, particularly in sub-Saharan Africa, Asia and Latin America [1, 24, 32, 53, 64, 65]. Among countries which report data on rehabilitation workers, ratios vary from <0.01 per 10 000 population in low-income countries to up to 25 per 10 000 population in high-income countries [1, 24, 25, 32, 53]. Only six physicians specialized in rehabilitation were identified in sub-Saharan Africa, all in South Africa [56].

Although scarcely studied, international migration appears to aggravate global inequalities: in the United States of America (USA), foreign-educated, recently licensed PTs came predominantly from the Philippines and India [66]. Singapore partly reduces shortages of rehabilitation workers by recruiting in resource-poorer Asian countries [25].

Among high-income countries, supply variability also exists [1, 24, 25]: Portugal has four times more PTs per capita than Singapore, whose GDP is three times higher [25]. Some ‘compensatory’ supply can exist across rehabilitation professions: in the USA, there are less per-capita PTs than in Portugal, but nearly twice the number of OTs. This reflects a partial role overlap, since many rehabilitation tasks (e.g. related to transfers, exercise) can be performed by PTs, OTs and other professionals (e.g. nurses, athletic trainers, PTs/OTs assistants) [25].

Few programs exist for educating qualified rehabilitation workers in lower income countries [1, 25, 56]. Alternative cadres (e.g. community-based rehabilitation workers), capable to work across sectors (health, social, educational) [67, 68], can partly mitigate that undersupply of health-specific rehabilitation workers, but the quantity and quality of the evidence on their effectiveness is currently scarce [68], and the initial intensity of instruction and supervision required are obstacles [67].

#### Accessibility

Access to rehabilitation services and workers is usually harder in rural or remote areas [69–71]. This includes high-income countries, such as the USA [26, 27, 60], Canada [72–75] and Australia [69, 70, 76–78]. Care rationing may come as a result [79]. Particularly in Canada [72–74] and Australia [68, 70, 76, 77, 80, 81], a set of educational, recruitment and retention measures have been implemented to help supply rural or remote areas with the rehabilitation workers they need.

In low-income countries, people in need are significantly challenged to access any rehabilitation health workers [1, 82]. Lack of transportation, physically inaccessible sites, inadequate equipment and service costs are other access barriers [1, 83–88].

Home [89], community [59, 88, 90] or tele-based [69, 81, 91] forms of rehabilitation care delivery increasingly are used to improve access to care in underserved areas.

In some high-income countries (e.g. USA, Australia, the United Kingdom), patient’s self-referral to rehabilitation workers has been increasingly implemented [92]. Besides promoting timely access to rehabilitation workers, it can achieve better outcomes at lower cost [93]. The model requires advanced therapists’ competencies (decide whether to treat or refer patients to physicians), which trained therapists have shown to possess [92, 94]. Such innovative model, as well as tele-rehabilitation, is however hampered by requirements of physician prescription (for third-party reimbursement), licensing and administrative barriers (on cross-state or cross-country delivery of tele-rehabilitation) or even lack of providers/patients’ knowledge that such option is available [25, 92].

Coverage gaps typically affect more the socially vulnerable people with disabilities, under- or uninsured, resource-poorest, belonging to disadvantaged race/ethnic groups and those living in rural or remote areas: this phenomenon of ‘double disparities’ accentuate the vicious circle of disability and social disadvantage [1, 95].

Finally, rehabilitation services delivered outside hospitals are typically less funded, less attractive to rehabilitation workers, financially and academically, and thereby less available to those in need [25, 96, 97].

#### Acceptability

Some initiatives report promoting culturally competent rehabilitation workers. These include studies focused on tailoring approaches to indigenous populations in Oceania [98, 99] or the local development of culturally relevant, community-based interventions for children with disabilities in Kenya [100]. Finally, some international clinical education/service placements, from higher to lower income countries, have overcome cultural implementation barriers [101–105]. The few other studies on cultural-competencies training within the literature have important methodological limitations (e.g. small samples, poor designs) [106].

The need for same-sex provider applies to rehabilitation in some cultures [1]. Female therapists, in turn, can be limited in traveling for training or in making home visits [1, 107, 108]. An authoritarian society, negative societal beliefs about disability, and the typical medical approach to treatment are other factors impeding the optimal delivery and demand for rehabilitation in lower income countries [1, 64, 65, 107, 108]. Finally, in lower income countries, particularly in rural communities, people might be unaware of rehabilitation and its benefits, thus reluctant to seek it, even when available [25, 108].

Understanding and changing families’ perceptions of disability and rehabilitation may enhance children with disabilities’ access to rehabilitation [109]. Community health workers, volunteers or key informants also can help assure that people with disabilities in those locations are either locally treated or appropriately referred to specialized/centralized rehabilitation centres [1, 110].

#### Quality

The competencies, skills and practices of rehabilitation workers can vary substantially across countries or practice locations.

PTs successfully take on advanced competencies such as ordering X-rays or making musculoskeletal ‘diagnosis’ in some jurisdictions within high-income countries (e.g. the United Kingdom, USA, Canada) [25, 111–113], but this can vary within countries (e.g. more in the military sector within the USA) [33, 114]. The same inter- and intra-national variability exists within educational requirements for licensure: 3-year clinical doctorates are increasingly required for PTs/OTs in the USA, but PTs/OTs can work with lower credentials [25, 115].

PTs/OTs with more advanced practices increasingly delegate high-volume, less-skilled tasks to PT/OT assistants [25, 116, 117], in countries where these exist [25]. This task-shifting has been effective particularly when well-planned, studied or enabled by supervision or supportive tools [118, 119], but can be detrimental to both costs and outcomes otherwise (e.g. PT assistants delivering care without appropriate supervision) [120].

Where demand clearly exceeds supply, a cross-disciplinary assimilation of practices occurs more among rehabilitation workers [1, 25, 59]. Also due the huge amount of unmet needs, in low-income countries, people with disabilities are often discharged rapidly, irrespective of recovery, for more people to be attended. This pressures therapists to deliver aggressive therapy, with unknown consequences for quality of care [25].

### Phase 2: integrating the reviewed information into a SWOT analysis framework

A SWOT analysis was made of the critical review results (Table 3).

Major strengths (S) relate to some specific research: the literature broadly identifies where higher unmet needs for rehabilitation health workers exist, e.g. in lower income countries, and in rural regions elsewhere. The literature also identifies distance education and international clinical education and service placements, from higher to lower income countries.

**Table 3:** The reviewed rehabilitation workforce literature integrated into SWOT analysis framework

Strengths • Unmet needs for rehabilitation workers are broadly identified: e.g. more in low-income countries and in rural regions elsewhere. • Already existing initiatives report promoting culturally competent rehabilitation, such as for aboriginal communities in Oceana and rural communities in sub-Saharan Africa. • Existence of long-distance education and international clinical education/service placements, inclusively from higher to lower income countries. • Existing knowledge of initiatives and factors that influence/improve the recruitment and retention of rehabilitation health workers in rural or remote areas of some high-income countries.	Weaknesses • No agreed strategy to determine rehabilitation supply requirements. • Under-development of information systems for monitoring supply. • Absence of professional registration/licensing/regulation for rehabilitation workers in many countries. • Lack of a uniform classification for different rehabilitation competencies, practices and credentials. • Lack of training programs for educating qualified rehabilitation workers in low-income countries. • International migration seems to aggravate global inequalities, but it has been scarcely studied. • Lack of physically accessible sites, inadequate equipment, lack of transportation and lack of capacity of people with disabilities to afford rehabilitation services impede access. • Existing barriers (e.g. legal, lack of funding or stakeholders’ awareness) that prevent access to rehabilitation care. • Coverage gaps typically affect more the socially vulnerable people with disabilities (under/uninsured, resource-poorest, belonging to disadvantaged race/ethnic groups, living in rural or remote areas). • Rehabilitation services delivered outside hospitals are typically less funded, less attractive to rehabilitation workers and thereby less accessible to people with disabilities.
Opportunities • Possibilities for a global scaling-up of some initiatives that aim to supply underserved areas with needed rehabilitation workers. • Locally tailored policy solutions and innovative service delivery models are increasingly used and tested to enhance access to rehabilitation in underserved areas. • Possible solutions to undersupply and inadequate skill-mix (e.g. shifting and sharing of competencies across rehabilitation workers and other health providers). • The study and development of the rehabilitation workforce can benefit from, and be integrated within, the recent advances in the field of Human Resources for Health toward universal health coverage.	Threats • Complexity and heterogeneity on the composition of the rehabilitation workforce. • Variability of competencies and scope of practice within the same professional label across countries and some within the same country. • Oversimplification: e.g. studying, monitoring and developing specific professions, nationally and internationally, instead of a whole rehabilitation workforce—including how this is distributed by regions, sectors, service-levels, etc. • Low priority in the health agenda.

Major weaknesses (W) are the sub-development of supply data sources and of monitoring mechanisms. A uniform, international classification for defining different competencies, constituents and practices of rehabilitation workers is lacking. Also, there is no agreed strategy to determine rehabilitation supply requirements.

Opportunities (O) include possibilities for global scaling-up of some exemplary initiatives, for example from Canada and Australia, aimed at attaining rehabilitation workers willing and capable of working in underserved areas. Locally tailored policy solutions (e.g. outreach programs) and innovative service delivery models like tele-rehab are also increasingly tested to enhance access in underserved areas. Solutions for undersupply and inadequate skill mix of rehabilitation workers (e.g. task-shifting, particularly when assisted; cross-disciplinary assimilation of practices) may be widely applicable.

Threats (T) emanate from the inherent complexity of the physical rehabilitation workforce, which may assume multiple configurations of professions, varying in education, competencies and practices, even within the same profession. The risk is to try to overcome this complexity by oversimplification, e.g. only monitoring specific professions instead of monitoring also the whole physical rehabilitation workforce. Finally, in the face of that complexity, and the low policy priority given to disability and rehabilitation, there is a risk of a continued negligence in the study and development of the rehabilitation workforce.

## Discussion

This section focuses on presenting six rehabilitation workforce challenges identified through the literature review and SWOT analysis. As a whole, identification of these challenges could advance the rehabilitation workforce agenda and inform researchers and policy-makers on advancing rehabilitation workforce studies and policies.

**Fig. 1:**
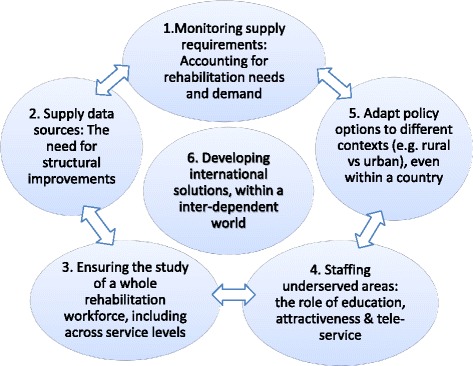
The Six Rehab-Workforce Challenges

The challenges are displayed in Fig. 1 as an inter-dependent whole, including a last, central element underpinning all others.

### Monitoring supply requirements: accounting for rehabilitation needs and demand

Many studies reviewed did not account for rehabilitation supply requirements beyond population size [1, 22, 32, 60]. When they did, either indicators of ‘need’ (e.g. epidemiological) [24–26] or ‘demand’ (e.g. services utilization, unfilled vacancies) [61–63] were used. Therefore, a first challenge is to globally debate and eventually agree on a standard method to assess rehabilitation supply requirements.

Toward that end, the concepts of ‘need’ (i.e. whether and how much the population require rehabilitation, derived from demographics and epidemiological data) and ‘demand’ (e.g. whether and how much rehabilitation services are actually sought and utilized by the population, regardless of underlying need) might be considered, with their relative pros and cons. For example, using demand requirements can lead to perpetuating systems’ inefficiencies, by allocating more resources where least needed. On the other hand, using need indicators may not account for whether population will actually use rehabilitation services, e.g. by lack of financial coverage or population unawareness that these resources are available [25, 108, 121–123].

Finally, the most suitable metrics and measures of need and demand to be used as rehabilitation supply requirements also need to be determined, inclusively considering recent practices and advancements in disability measurement [124–126].

### Supply data sources: the need for structural improvements

Studies commonly report important limitations in the availability of accurate, reliable, comprehensive, disaggregated (by profession and working sector) and comparable supply data [1, 24, 32]. Apart from being a low priority, the monitoring of this workforce is complicated by the lack of common definitions of who are rehabilitation health workers and the lack of classifications that accommodate the varying competencies and practices within the same profession, both within and across countries [24, 50]. When available, rehabilitation workforce data is often aggregated illogically, e.g. mixing rehabilitation workers with unrelated health professions [51].

The monitoring of the rehabilitation workforce would need (i) investments in the collection and analysis of national rehabilitation workforce data, facilitated by the utilization of a minimum data set and registration of practitioners; (ii) agreed professional definitions, classifications and credentials; and (iii) improved availability of data, in formats that do not aggregate rehabilitation health workers with other occupational groups. For any international comparisons, definitions and classifications should be globally standardized, as much as feasible.

### Ensuring the study of the whole rehabilitation workforce, including across service levels.

The rehabilitation workforce is principally composed of professionals specifically trained to provide rehabilitation care, but many other health workers, even non-health workers, sometimes meet the physical rehabilitation needs of the population. Examples are physicians, nurses, community health workers, athletic trainers and even special education teachers.

When meeting those needs, all these groups, within their specific competencies and practices, need to be considered when estimating the supply of rehabilitation workers.

Finally, for a comprehensive determination of the rehabilitation workforce and of the unmet needs, studies can include how this workforce is allocated across employment sectors (public vs private; health vs educational/social), the healthcare continuum (primary, acute, post-acute, long-term care) and practice locations (inpatient, outpatient, home-based, community-based) [25, 71, 96, 97].

### Staffing underserved areas: the role of education, attractiveness and tele-service

Lower income countries and rural and remote regions are typically underserved by health workers and more so by rehabilitation health workers. Policy options to address this challenge may include a number of possibilities which can and should be mutually complementary.

First, some rehabilitation education programs might focus on the competencies for working in underserved contexts, while clinical education and field experience in those locations are also useful [59, 73, 75, 78, 101–103, 111]. This can be complemented by attracting students from underserved regions, through a mix of financial (scholarships, stipends) and non-financial incentives (in-kind benefits, mentorship) [1, 72, 73, 78, 80].

Second, for those already working in underserved locations, it is important to design and implement retention measures, like opportunities for distance learning, career development and support systems [70]. Other examples are financial incentives, service arrangements for spending few nights away, and support for accommodation, the education of children and integrating spouses in the labour market [76, 127, 128].

A third option is to develop remote services delivered by tele-means [25, 69, 81, 91, 129], even from outside the country. This strategy can also be used for training students, their educators [130–133] and even coaching frontline staff (peer-professionals, lower-level rehabilitation workers), volunteers or family members [25, 134].

Finally, health workers such as physician, nurses or community health workers also can be trained to acquire and apply rehabilitation competencies within their practices [1, 41, 67, 68, 135, 136].

Currently, few examples exist for any of those initiatives, and encouraging countries to introduce them is a major challenge.

### Adapt policy options to different contexts (e.g. rural vs urban), even within a country

Meeting rehabilitation workforce needs, and ultimately the population rehabilitation needs, may require different solutions for not only different countries but also within a country (rural/remote vs metropolitan regions) [1, 25, 41, 59].

The key challenge in ‘well-served’ areas (e.g. metropolitan areas in high-income countries) is to create conditions for the entire rehabilitation workforce to perform at the top of their credentials, to achieve the best outcomes at the lowest cost.

That may include policy action such as (i) designing and implementing mechanisms (outcome monitoring, value-based-reimbursement) for higher accountability for the value of care [11]; (ii) removing barriers (e.g. need for physician prescription, lack of third-party reimbursement; licensing barriers to cross-state delivery) hampering implementation of innovative, cost/supply-effective models of rehabilitation care access and delivery (e.g. direct access to rehabilitation therapists [92, 93], tele-rehabilitation [91, 129]); and finally (iii) supporting task-shifting processes such as therapists taking on some advanced care roles [25, 111–113], while rehabilitation tasks of high volume but low skill are transferred to providers of lower education and cost [25, 116].

All of this can be productive if well-planned, studied and supported by adequate training, supervision or decision-making algorithms [118, 119].

Savings from efficiency gains in ‘well-served’ locations can be re-allocated to ensure that the more vulnerable population has access to the rehabilitation health workers they need [25, 33, 137].

While policies for universal rehabilitation coverage might be in place, supplying underserved areas may also require implementing (i) trans-disciplinary models of rehabilitation practice [75, 77, 111]; (ii) more accessible, locally shaped forms of rehabilitation service delivery (community-, home-, tele-based) [59, 69, 77, 81, 100] and finally (iii) response outreach programs that cut across institutional (public, private, NGOs) and traditional healthcare silos [69, 77, 81, 138, 139].

### Developing international solutions, within a inter-dependent world

While locally tailored solutions are certainly appropriate, that does not mean that global, integrative solutions do not apply.

For instance, international migration of rehabilitation workers and its determinant have been under researched. Such studies are best achieved when using data from both sending and receiving countries. Besides, policies may aim to take benefit from international mobility, instead of just mitigating potential perverse effects of the so-called brain drain [34, 127].

For example, international clinical placements and temporary exchange programs of clinicians and students, inclusively among countries of high and low income [101, 102, 104, 140], can bring benefits on both sides of the table: service/knowledge for where it is most needed and learned competencies applied back home [103, 140–142]. All of this requires, however, overcoming any applicable cultural, financial and operational barriers to program implementation [101, 105]. Ultimately, such international exchange helps educating rehabilitation workers capable of working, advocating, researching and thinking globally [143, 144].

Finally, international health technical aid is required on a regular basis [1], but more in humanitarian crises created by natural disasters or armed conflicts, which exponentially increase physical rehabilitation needs [82, 143, 145, 146].

### Limitations

Although using a structured PubMed search, the critical review does not reflect a systematic or scoping review approach, so these results should not be understood as such. Besides, exclusion of non-English papers and the absence of a structured search for the grey literature can turn the literature reviewed under-representative of certain countries’ scenarios (e.g. Latin America countries). Also, to provide a notion of the range of findings on each point being made, we often provide examples from high- and low-income countries. This does not obviate the need to study and develop the many countries’ realities in between.

## Conclusions

Global development policies, such as the Sustainable Development Goals, aim to improve the lives of marginalized groups, by reducing the burden of diseases and poverty. Meeting the rehabilitation needs of people with disabilities who are marginalized, have lower health status, lower healthcare access, often live in poverty and are limited in their social functioning would contribute to all these goals. Inspired by the Six Rehab-Workforce Challenges, which seem aligned with the global strategy on human resources for health [147], action from local and global policy-makers should to be taken.

Local policy-makers might (i) determine the local needs and/or demand for rehabilitation; (ii) develop mechanisms to monitor the available rehabilitation workforce, including across geographies and service levels; (iii) take action to reduce needs-based shortages of rehabilitation workers (e.g. implementing recruitment and retention measures, invest in local capacity building); and finally (iv) promoting rehabilitation systems strengthening, including the implementation of cost- and supply-effective service delivery models (e.g. tele- and community-based rehabilitation, direct access to well-trained therapists) and response outreach programs cutting across traditional silos.

Global policy-makers might (i) assure that more resources are allocated, equitably, to the study and development of the rehabilitation workforce; (ii) define international standards for assessing rehabilitation needs; (iii) develop uniform classifications of rehabilitation workers, competencies and practices; and finally (iv) support partnerships across countries for scaling-up local training and even for the cross-national service provision.

Lastly, human resources for health researchers and their funders must include rehabilitation workers in their agenda. If this is not done, the growing advances in the rehabilitation science and practice [43, 148] will continue to be unavailable to those who most need them.

## Additional files

Additional file 1: Search strategy in PubMed. (DOCX 14 kb)
Additional file 2: Papers excluded within the synthesis stage, and the reasons to do so. (DOCX 20 kb)
Additional file 3: Data extraction table. (DOCX 111 kb)

## Abbreviations

AAAQ: Availability, accessibility, accessibility and quality
GDP: Gross domestic product
NGOs: Non-government organisations
OTs: Occupational therapists
PTs: Physical therapists
SWOT: Strengths, weaknesses, opportunities and threats
