# Supplementary material for: Human resources for health (and rehabilitation): Six Rehab-Workforce Challenges for the century
Source: Hum Resour Health. 2017 Jan 23;15:8. doi: 10.1186/s12960-017-0182-7 (PMC5259954; doi:10.1186/s12960-017-0182-7)
Supplement: Additional file 1: — Search strategy in PubMed. (DOCX 14 kb) [file 12960_2017_182_MOESM1_ESM.docx]

**Appendix 1**

**Search strategy in PubMed**

("manpower"[All Fields] OR "Health Manpower"[All Fields]) AND ("rehabilitation"[Subheading] OR "rehabilitation"[All Fields] OR "rehabilitation"[MeSH] OR "Physical and Rehabilitation Medicine"[Mesh] OR "Rehabilitation of Speech and Language Disorders"[Mesh] OR "rehabilitation centers"[MeSH] OR "Rehabilitation Nursing"[Mesh] OR "Physical Therapists"[Mesh] OR "Occupational Therapy"[Mesh] OR "Speech-Language Pathology"[Mesh] OR "Rehabilitation, Vocational"[Mesh] OR "Activities of Daily Living"[Mesh] NOT "Correction of Hearing Impairment"[Mesh] NOT "Substance Abuse Treatment Centers"[Mesh] NOT "Mouth Rehabilitation"[Mesh]) AND ("2006/03/31"[PDAT] : "2016/03/31"[PDAT])

**Note:**

- The organization of the MeSH tree and the detailed definitions and elements included within each MeSH term can be found and searched online at: <http://www.ncbi.nlm.nih.gov/mesh/1000048>.
