# Supplementary material for: Human resources for health (and rehabilitation): Six Rehab-Workforce Challenges for the century
Source: Hum Resour Health. 2017 Jan 23;15:8. doi: 10.1186/s12960-017-0182-7 (PMC5259954; doi:10.1186/s12960-017-0182-7)
Supplement: Additional file 2: — Papers excluded within the synthesis stage, and the reasons to do so. (DOCX 20 kb) [file 12960_2017_182_MOESM2_ESM.docx]

| Main reason | Paper | Specific Reason |
| --- | --- | --- |
| More specific papers exist within the (sub-)topic addressed | O'Brien R, Byrne N, Mitchell R, Ferguson A.  Rural speech-language pathologists' perceptions of working with allied health assistants.  Int J Speech Lang Pathol. 2013 Dec;15(6):613-22. | Only partly related. It refers to the perceptions of working alongside assistants, not specifically related for example to the study of a task shifting process and its outcomes, as other studies have analyzed. |
|  | Tillard GD.  Factors contributing to the brain drain in speech-language pathology: a New Zealand example.  Int J Speech Lang Pathol. 2011 Aug;13(4):360-8. | It focus only in the intentions to migrate of undergraduate students and recent graduates (hypothetically in the future), not the underlying factors within actual migration. |
|  | Cocks N, Cruice M.  The experiences and perspectives of overseas trained speech and language therapists working in the United Kingdom.  Int J Speech Lang Pathol. 2010 Jun;12(3):271-82. | The aimed to explore the experiences and perspectives of migrant speech and language therapists living in the UK, but the conclusions only report findings very aligned any other migrant health workers. |
|  | Norman KE, Booth R, Chisholm B, Ellerton C, Jelley W4, Macphail A, Mooney PE, Mori B, Taipalus L, Thomas BK.  Physiotherapists and Physiotherapy Student Placements across Regions in Ontario: A Descriptive Comparison.  Physiother Can. 2013 Winter;65(1):64-73. | It describes the distribution and type of physiotherapy student placements in one year relative to the number of practicing physiotherapists of Ontario.  However, it does not directly focus on the distribution by regions of providers themselves and does not delve into the underlying factors affecting those distributions. |
|  | Harwood M, Weatherall M, Talemaitoga A, Barber PA, Gommans J, Taylor W, McPherson K, McNaughton H.  Taking charge after stroke: promoting self-directed rehabilitation to improve quality of life--a randomized controlled trial.  Clin Rehabil. 2012, Vol. 26(6), pp. 493-501. | It includes Maori and Pacific New Zealanders as participations, but the primary focus is promoting self-directed rehabilitation while there are others primarily focused on tailoring approaches to indigenous populations of Oceania. |
|  | McLaughlin EG, Adamson BJ, Lincoln MA, Pallant JF, Cooper CL.  Turnover and intent to leave among speech pathologists.  Aust Health Rev. 2010 May;34(2):227-33. | It focuses only in the intentions to migrate (hypothetically, in the future) and the profile of those intending to leave, not the underlying factors within actual migration. |
|  | O'Toole K, Schoo AM.  Retention policies for allied health professionals in rural areas: a survey of private practitioners.  Rural Remote Health. 2010 Apr-Jun;10(2):1331. Epub 2010 May 3. | It explores the thoughts and perceptions of private rehabilitation therapists in rural areas particularly about an agreement/disagreement with partnerships between private and public practice in rural and regional areas, or even the use of government incentives to retain and attract private practitioners to rural and regional areas.  There is no assessment of effectiveness of any of those policies in practice. |
| Published before 2008 | Pitblado JR. 2007  Rural net migration of Canada's rehabilitative and technical health care workers.  Cah Sociol Demogr Med. 2007 Oct-Dec;47(4):427-44 | Published in 2007, within a topic and context addressed by more recent articles. |
|  | Whitehouse AJ, Hird K, Cocks N.  The recruitment and retention of speech and language therapists: what do university students find important?  J Allied Health. 2007 Fall;36(3):131-6. | Published in 2007, within a topic addressed by more recent articles. |
|  | Jette DU, Ardleigh K, Chandler K, McShea L.  Decision-making ability of physical therapists: physical therapy intervention or medical referral.  Phys Ther. 2006, Vol. 86(12), pp. 1619-29. | Published in 2006, while there are more recent studies included addressing the topic and coming to similar conclusions. |
|  | Brusco NK, Shields N, Taylor NF, Paratz J.  A Saturday physiotherapy service may decrease length of stay in patients undergoing rehabilitation in hospital: a randomised controlled trial.  Aust J Physiother. 2007;53(2):75-81. | Even though it relates with timely access to rehabilitation services, it was published back in 2007 - and it is not certain whether the issue addressed is a relevant problem today. |
|  | Dunleavy, K.  Physical therapy education and provision in Cambodia: a framework for choice of systems for development projects.  Disabil Rehabil. 2007, Vols. 29(11-12), pp. 903-20 | Published in 2007. There are more recent papers addressing the education and delivery of physical therapy in low-income Asian countries. |
|  | Tinney MJ, Chiodo A, Haig A, Wiredu E.  Medical rehabilitation in Ghana.  Disabil Rehabil. 2007, Vols. 29(11-12), pp. 921-7. | Published in 2007. There are more recent papers addressing the access to and supply of rehabilitation services and workers in Africa. |
|  | Wolf-Branigin M, Wolf-Branigin K, Israel N.  Complexities in attracting and retaining direct support professionals.  J Soc Work Disabil Rehabil. 2007;6(4):15-30. | Published in 2007, within a topic which is indirectly related to rehabilitation (i.e. personal assistants or direct support worker for the inclusion of people with disabilities). |
|  | Stanmore E, Waterman H.  Crossing professional and organizational boundaries: the implementation of generic rehabilitation assistants within three organizations in the northwest of England.  Disabil Rehabil. 2007, Vol. 29(9), pp. 751-9. | Published in 2007. There are more recent papers referring to the development of the role of mid-level assistants. |
|  | Stanmore E, Ormrod S, Waterman H.  New roles in rehabilitation--the implications for nurses and other professionals.  J Eval Clin Pract. 2006 Dec;12(6):656-64. | Published in 2006. There are more recent papers referring to the development of the role of mid-level assistants. |
| Dropped in face of more recent systematic reviews | Boshoff K, Hartshorne S.  Profile of occupational therapy practice in rural and remote South Australia.  Aust J Rural Health. 2008 Oct;16(5):255-61. | Results included in the systematic review with meta-synthesis from Roots et al |
|  | Aiken, A  Improved use of allied health professionals in the health care system: the case of the advanced practice physiotherapist in orthopedic care.  World Hosp Health Serv. 2012;48(1):28-30. | Excluded in face of the inclusion of recent systematic reviews (i.e. Desmueles et al; Oakley et al) within the topic of advanced care practices |
|  | McAuliffe T, Barnett F.  Perceptions towards rural and remote practice: a study of final year occupational therapy students studying in a regional university in Australia.  Aust Occup Ther J. 2010 Oct;57(5):293-300. | It focuses in the student’s career intentions. There is another, contemporary study from the same authors (included in our results) reviewing the literature on the topic. |
