# Supplementary material for: Human resources for health (and rehabilitation): Six Rehab-Workforce Challenges for the century
Source: Hum Resour Health. 2017 Jan 23;15:8. doi: 10.1186/s12960-017-0182-7 (PMC5259954; doi:10.1186/s12960-017-0182-7)
Supplement: Additional file 3: — Data extraction table. (DOCX 111 kb) [file 12960_2017_182_MOESM3_ESM.docx]

| **Cited as no.** | **Category**  **(AAAQ )** | **(Sub-)Topics Addressed:**  **justifying inclusion** | **Author, title & source** | **Summary of papers’ goals and methods** | **Main information retrieved**  **(e.g. abstracted from results and/or conclusions)** |
| --- | --- | --- | --- | --- | --- |
| 1 | Availability, Accessibility, Acceptability, Quality | - Supply and need for rehabilitation; - Distribution of rehabilitation workers; - Characteristics of rehabilitation services and workers all around the globe | World Health Organization.  World Report on Disability.  Geneve : WHO, 2011. | - This landmark report from the World Health Organization refers to the greatest collection of evidence related to disability and its health-related aspects, including rehabilitation. - The evidence was compiled by a large group of experts in the field - “Rehabilitation” was the main chapter consulted. | - Estimated 1 billion of people worldwide with disability, about 80% living in lower income countries. - Global data on the need for rehabilitation ser­vices do not exist. Data on rehabilitation services are often incomplete and fragmented. When data are available, com­parability is hampered by differences in defini­tions, classifications of measures and personnel, populations under study, measurement meth­ods, indicators, and data sources. - The need for rehabili­tation is projected to increase due to demographic and epidemiological factors. - Global information about the rehabilita­tion workforce is inadequate. Supply data from countries who report data vary from <0.01 to 21 (in Finland) PTs per 10,000 population and from <0.01 to 11 (in Denmark) OTs per 10,000 population. - It points for action needed, particularly in lower income countries: addressing barriers to rehabilitation; reforming policies, laws and delivery systems; establishing national rehabilitation plans and (international) collaborations; developing funding mechanisms for rehabilitation - Specifically points for the need to: increase the human resources for rehabilitation; expanding education and training; training existing healthcare personnel for rehabilitation; building training capacity; contextualizing the curricula content; paying attention to the recruiting and retention; expanding and decentralizing service delivery; increasing the use and affordability of technology, including tele-rehabilitation; expanding research and evidence-based practice – based on accurate data. |
| 24 | Availability | - International supply of rehabilitation workers (quantification) - Supply in relation to need indicators - Workforce data sources | Gupta N, Castillo-Laborde C, Landry MD.  Health-related rehabilitation services: assessing the global supply of and need for human resources.  BMC Health Serv Res. 2011 Oct 17;11:276. | - To quantitatively describe the global supply of and need for human resources for health-related rehabilitation services. - Data for assessing supply of and need for rehabilitative personnel were extracted and analyzed from statistical databases maintained by the WHO and other (inter)national health information sources. | - Large differences (e.g. allied health workers associated to rehabilitation: from <0.01 (many low-income countries) to 25 (Finland) per 10,000 habitants) were found across countries and regions between assessed need for services requiring health workers associated to physical and rehabilitation medicine against estimated supply of personnel skilled in rehabilitation services. - Despite greater need, low- and middle-income countries tended to report less availability of skilled health personnel. - The evidence base remains fragmented; the result of limited availability and use of quality, comparable data and information within and across countries. |
| 25 | Availability, Accessibility Quality | - International supply of rehabilitation workers (comparison) - Supply in relation to need indicators - Workforce data sources - Access to rehabilitation - Scope of practice | Jesus T, Koh G, Landry M, Ong P, Green P, Lopes A, Hoenig H.  Finding the ‘Right Size’ Supply of Physical Therapists: An international perspective across four countries.  Phys Ther. 2016, [Epub ahead of print] | - It paper provides a comprehensive examination of PT workforce issues across 4 distinct countries (United States, Singapore, Portugal and Bangladesh), deliberately selected to allow consideration of key contextual factors. - This investigation provides a theoretical model uniquely adapted to focus on variables most likely to affect PT workforce needs. This theoretical model was used to guide acquisition of public domain data across the respective countries. - It provides a contextualized interpretation about the PT workforce supply. | - PTs (and new nationally trained PTs: 1995-2014)per 10,000 population: Bangladesh <0.1 (0.09); Singapore 1.8 (1.54); United States 6.5 (4.4); Portugal 7.8 (8.3). - Bangladesh has the highest unmet need for PT and the lowest number of educational programs. - Access barriers (financial, organizational) exist across the countries. - The few patients receiving centralized PT in Bangladesh typically are discharged shortly. This places pressures on PTs to deliver aggressive therapy. - In Singapore, relative shortages are partly compensated by immigration, at the cost of neighboring lower-income countries. - The lower PT supply of the United States relative to Portugal is compensated by nearly 2 times more OTs in the United States. - Plasticity in the workforce is reflected in the scopes of practices. For example, training in transfers and activities of daily living can be provided by PTs, OTs, and nurses across the countries. - Advanced education and care competencies were present particularly in the US. - There may not be a 'one-size-fits-all' recommendation for PT workforce supply, its determination and development. |
| 26 | Availability, Accessibility | - Analysis of supply in relation to age and population size (United States and their individual states). - Forecasting of the Physical Therapy supply. - Data sources | Zimbelman JL, Juraschek SP, Zhang X, Lin VW.  Physical therapy workforce in the United States: forecasting nationwide shortages.  PM R. 2010 Nov;2(11):1021-9. | - To examine current and future physical therapy (PT) job surplus/shortage trends across the United States. - Forecast models and grading methodology previously published for nursing were used to evaluate individual state PT job shortages from 2008 to 2030. - The forecast model used to project PT job supply and demand accounted for changes in age and population size on the basis of estimates from the U.S. Census Bureau for each of the 50 states. | - On the basis of current trends, demand for PT services will outpace the supply of PTs within the United States. - Shortages are expected to increase for all 50 states through 2030. - By 2030, the number of states receiving below-average grades for their PT shortages will increase from 12 to 48. - States in the Northeast are projected to have the smallest shortages, whereas states in the south and west are projected to have the largest shortages. - The model has several limitations and may be oversimplified. |
| 32 | Availability | - Supply of PTs by country. - National data sources | Sykes C, Bury T, Myers B.  Physical therapy counts: counting physical therapists worldwide.  BMC Health Services Research. 2014, Vol. 14(Suppl 2), p. O23 . | - To fill the information gap, the World Confederation for Physical Therapy (WCPT) has developed an annual data collection which generates a country profile that includes information on the WCPT member organisations (MOs), the number of PTs, scope of practice, education and regulation. - Where available, international standard classifications were used. - An online data capture tool was built and pilot tested. The first collection was made in 2012. | - In 2012, 69 of 106 WCPT MOs provided data, a 65% response rate. - The analysis enabled reporting on numbers of PTs who were members of WCPT MOs and an estimate of the number of PTs in the country of the responding MOs, which paired with population data from the World Bank provided an estimate of the PT to population ratio. - The ratio varied between 0.002 PTs per 1000 population to 2.82 per 1000 population. - The collection is in its infancy and data quality and reporting requires improvement. |
| 50 | Availability | - Operational boundaries of the rehabilitation workforce - Supply data sources | World Health Organization.  Monitoring human resources for health-related rehabilitation services. Spotlight on health workforce statistics.  Geneve : WHO, 2009. Available at: http://www.who.int/hrh/statistics/spotlight_7_en.pdf | - Use of data sources available to the World Health Organization. | - In many countries, at the global level, information on human resources for rehabilitation is inadequate and fragmented. This is partly related to lack of common definitions and classifications, partly to poor availability and use of standard statistical sources for workforce monitoring, and partly to lack of political will to place monitoring of HRH for rehabilitation high on the health agenda – the latter itself related to the way societies often interpret and react to disability. - Strengthening the global information base on the different health occupations based on timely, comprehensive and reliable data is prerequisite for evidence-informed workforce development strategies in rehabilitation. - Information on the rehabilitation workforce within and across countries and over time is the setting of common definitions and classifications of who are rehabilitation health workers. - There is no single operational boundary of what constitutes the rehabilitation health workforce |
| 51 | Availability | - Global supply of rehabilitation personnel - (Inter)national Data sources, display and organization | World Health Organization.  Global Atlas of the Health Workforce. [Online].  Available in different formats from: <http://apps.who.int/gho/data/node.main?showonly=HWF>. Accessed: March 30, 2016 | - Based on (inter)national data sources on the availability of health personnel, organized by varied categories. - The method of estimation for numbers of “other health service providers” depends on the organization of the national health system and the nature of the original data source. - WHO compiles data on health workforce from four major sources: population censuses, labour force and employment surveys, health facility assessments and routine administrative information systems (including reports on public expenditure, staffing and payroll as well as professional training, registration and licensure). | - Health workers skilled in rehabilitation are framed within a group of other health service providers, which include a large range of occupations such as ambulance workers, dieticians and nutritionists, environmental and occupational health inspectors, medical assistants, medical imaging technicians, medical laboratory technicians, optometrists, paramedical practitioners, personal care workers, pharmaceutical personnel, physiotherapists, speech therapists, and traditional and complementary medicine practitioners. - Data exists specifically for: Physicians; Nursing and midwifery personnel; Dentistry personnel; Pharmaceutical personnel; Laboratory health workers; Medical assistants; Community and traditional health workers; Personal care workers; Biomedical engineers, etc – but not for rehabilitation health workers. |
| 52 | Availability | - International Migration - Data Sources | Pittman P, Frogner B, Bass E, Dunham C.  International recruitment of allied health professionals to the United States: piecing together the picture with imperfect data.  J Allied Health. 2014, Vol. 43(2), pp. 79-87 | - Research on the international recruitment of health professionals to the U.S. has focused almost exclusively on physicians and nurses; authors are aware of no research on the migration of allied health professionals. - The study examined the strengths and weaknesses of various public and private data sources on foreign-educated allied health professions in the U.S. and patched together a picture of these migrants. - The study was focused on pharmacists, physical therapists (PTs), occupational therapists (OTs), speech language pathologists (SLPs), and medical and clinical laboratory technicians (lab techs). | - 12% of PTs, 12% of lab techs, 8% of pharmacists, 4% of OTs, and 3% of SLPs are foreign-born and entered the U.S. at age 21 or older. - Among foreign-born PTs, about half remain as non-citizens, suggesting the highest proportion of recent arrivals among the five professions. - The development of better immigration data, disaggregated by occupation, is suggested. |
| 53 | Availability | - Number of practicing OTs by country, in relation to population size. - Profile of OTs | World Federation of Occupational Therapists.  WFOT Human Resources Project 2014, Edited Version .  Available from: http://www.wfot.org/ResourceCentre.aspx : WFOT, 2014.  Accessed, December 20, 2015 | - It collects data on Number of OTs and other national workforce information available such a number of training programs for OTs, number of OTs who are members of their professional associations, etc. - Number of WFOT Member Organisations surveyed = 73 - Number of WFOT Member Organisations that responded = 72 Response rate = 99%. - Non WFOT Member Organisations that provided data about the number of practising occupational therapists; Kosovo, Lesotho, Madagascar, Malawi, Rwanda, Zambia - Date questionnaire circulated: 15th October 2013. - Data collection method - Excel Spreadsheet circulated by WFOT Secretariat by email. Compiled and designed centrally | - Among data collected, 34 countries are identified with major shortages of OTs. This includes lower income countries in Africa, Latin America and Asia, but also some higher income countries such as Portugal, New Zealand, Australia, France, Hong Kong and Singapore. - There are 1106 OT education programs identified. - Number of occupational therapists per 10,000 head of population = median 0.9, mean 2 - In Kosovo, Madagascar and Rwanda, only one OT was identified. - Percentage of occupational therapists that are female = 83% Average percentage of occupational therapists working in government/public funded posts = 63% Average percentage of occupational therapists working in non government/public funded posts = 33% - Not all organisations collate data that was requested for this project. Missing data should not be interpreted as being not applicable; in some instances the information is not known or unable to be provided. |
| 54 | Availability | - Supply of OTs; - Education programs | World Federation of Occupational Therapists.  Developing, Occupational Therapy profession in countries which are not yet member of the WFOT… A Resource Package.  Forrestfield:WFOT; 2008. | - Resource package from the World Federation of Occupational Therapists | - It rovides key information from the World Federation of Occupational Therapists and the assistance it can provide to the many countries who do not have health workers from the occupational therapy profession and which seeks to introduce the profession |
| 55 | Availability, Quality | - Supply data sources - Practice regulation | The World Health Professions Alliance.  Regulation a top priority agree global health professionals.  WPA, Geneva: 2014.  Available at: http://www.whpa.org/Press_release_WHPA_Regulation_21May2014.pdf. | - Position paper at the aftermath of The World Health Professions Regulation Conference 2014 was held in Geneva, Switzerland on 17th and 18th May. Its title was: “Health professional regulation – facing challenges to acting in the public interest”. - The World Health Professions Alliance (WHPA) brings together the following organisations: the International Council of Nurses, the International Pharmaceutical Federation, the World Confederation for Physical Therapy, the World Dental Federation and the World Medical Association. | - Participants agreed that different systems suit different national environments, but whatever the model, regulation is a responsibility and a public duty, not an option. - They said that regulation systems should be underpinned by accountability and responsiveness and should observe principles such as checks and balances between stakeholders, and patients and professionals being aware of rights and duties. - The President of the World Confederation for Physical Therapy said: “It is clear that there is no single model for a good regulatory system, but all should ensure that physical therapists and other health professionals provide safe and competent care/services. - Regulatory bodies also need to understand the day-to-day realities of the health professions they are seeking to regulate.” |
| 56 | Availability | - Supply of African physiatrists - Data sources | Haig AJ, Im J, Adewole A, Nelson VS, Krabek B.  The practice of physical medicine and rehabilitation in sub-Saharan Africa and Antarctica: a white paper or a black mark?  Disabil Rehabil. 2009;31(13):1031-7. | - To explore the practice of PM&R in subSaharan Africa and Antarctica. - Medline searches, membership data searches, fax survey of medical schools, Internet searches, and interviews with experts. - The use of multiple methods aims to overcome the lack of common and/or reliable data sources. | - SubSaharan Africa has no PM&R training programs, no professional organizations, no specialty board requirements, and no practicing physicians in the field. - The 788,000,000 permanent residents of subSaharan Africa including approximately 78 million persons with disability are left unserved. - Contact with the South African Medical Association revealed that there were indeed 6 physicians in the country who specialized in PM&R – but none registered for example in the international society of these specialists. - Number of Physiatrists (data compared): There are over than 10280 in Europe; 7000 in the US, and about 10000 in China. - Development spends billions of dollars to support Africa; but aside from the small Lehay War Victims Fund, almost no investment in rehabilitation. - Some physiatrist volunteers donate their time, but then go home. Permanent solutions need to exist. - Africa is in a crisis. Local medical schools, hospitals doctors, and persons with disability; along with foreign volunteers, aid groups, and policymakers can impact the crisis. |
| 57 | Availability | - Data sources - Professional registration & regulation | World Federation of Occupational Therapists.  Position Statement: Professional Registration.  Available from <http://www.wfot.org/ResourceCentre.aspx>. Accessed January, 13,2016 | - Position statement | - The mechanisms of practice registration and regulation vary widely across countries - Professional regulation or registration, no matter its structure, affords the occupational therapy profession greater authority, control, and professional prestige. |
| 58 | Availability, Quality | - Availability of Physical therapists in Critical Care Units (Sri Lanka, compared with the literature in other countries) - Profile and scope of practice, and (continuing) education. | Sigera PC, Tunpattu MU, Jayashantha TP, De Silva AP, Athapattu PL, Dondorp A, Haniffa R.  National Profile of Physiotherapists in Critical Care Units of Sri Lanka: A Low-Middle Income Country.  Phys Ther. 2016, Vol. [Epub ahead of print]. | - To determine: (1) the availability of critical care physical therapist services, (2) the equipment and techniques used and needed, and (3) the training and continuous professional development of physical therapists. - All physical therapists working in critical care units (CCUs) of state hospitals in Sri Lanka were contacted. The study tool used was an interviewer-administered telephone questionnaire. - The response rate was 100% (N=213). | - Ninety-four percent of the respondents were at least diploma holders in physical therapy, and 6% had non-physical therapy degrees. - Most (n=145, 68%) had engaged in some continuous professional development in the past year. The majority (n=119, 56%) attended to patients after referral from medical staff. Seventy-seven percent, 98%, and 96% worked at nights, on weekends, and on public holidays, respectively. - Lack of specialist training, lack of adequate physical therapy staff numbers, a heavy workload, and perceived lack of infection control in CCUs were the main difficulties they identified. - The availability of physical therapist services in CCUs in Sri Lanka, a lower middle-income country, was comparable to that in high-income countries, as per available literature, in terms of service availability and staffing, although the density of physical therapists remained very low, critical care training was limited, and resource limitations to physical therapy practices were evident. |
| 59 | Availability, Accessibility, Quality | - Supply of PTs - Rural models of care - Community-based rehabilitation: enhancing access to. - Scope of competencies and practice | Nualnetr, N.  Physical therapy roles in community based rehabilitation: a case study in rural areas of north eastern Thailand.  Asia Pacific Disability Rehabilitation Journal . 2009, Vols. 20(1):73-82 | - This action research aimed to explore how physical therapists could enhance the quality of life for persons with disabilities via a community-based rehabilitation (CBR) strategy. - The study was conducted in two rural sub-districts in northeastern Thailand. In each sub-district, several group meetings were arranged for persons with disabilities and their families, and various community members. - Participants were encouraged to discuss their perception of problems of the current rehabilitation services for persons with disabilities. Strategies to manage all problems were collaboratively identified and were implemented in order of priority according to the importance of the problem. - The outputs of CBR were evaluated by interviews and observation. | - Physical therapists had numerous roles in CBR, depending on the community’s circumstances. - The role of physical therapists was different between the CBR programmes of the two study areas. In sub-district A, the researchers mainly acted as the programme facilitators, meanwhile, acting as programme facilitators as well as skill trainers in sub-district B. This difference in finding may be due to a difference in the context of a CBR programme in each sub-district. - Although a physical therapist was available in the sub-district B’s community hospital, she hardly participated in the programme due to lack of time from her routine work in the hospital. Then, as physical therapists, it was inevitable that the researchers provide skill training for the volunteers. At the end of the study, however, the supervising role for village volunteers was transferred from the researchers to the community hospital’s physical therapist. - Examples of physical therapy roles in CBR may include instigators of CBR services, team leaders and managers, providers of direct care, and advisers to governments and local communities, on establishing CBR programmes. - There is no one model of CBR that will suit all circumstances. It is required is a needs-based activity developed in response to local circumstances. Thus, physical therapists need a high degree of flexibility and innovative thinking, and a wide range of management, practice, teaching and research skills, if they are to contribute effectively to CBR. - The preparation of such physical therapists requires development of a more client-centered community-oriented education programme. |
| 60 | Availability | - Supply of PTs - Cross-national comparison | Landry MD, Ricketts TC, Fraher E, Verrier MC.  Physical therapy health human resource ratios: a comparative analysis of the United States and Canada.  Phys Ther. 2009 Feb;89(2):149-61. | - Health human resource (HHR) ratios are a measure of workforce supply and are expressed as a ratio of the number of health care practitioners to a subset of the population. - In this study, HHR ratios for physical therapists across the United States were estimated in order to conduct a comparative analysis of the United States and Canada. - National US Census Bureau data were linked to jurisdictional estimates of registered physical therapists to create HHR ratios at 3 time points: 1995, 1999, and 2005. These results then were compared with the results of a similar study conducted by the same authors in Canada. | - he national HHR ratio across the United States in 1995 was 3.8 per 10,000 people; the ratio increased to 4.3 in 1999 and then to 6.2 in 2005. - The aggregated results indicated that HHR ratios across the United States increased by 61.3% between 1995 and 2005. In contrast, the rate of evolution of HHR ratios in Canada was lower, with an estimated growth of 11.6% between 1991 and 2005. - Although there were wide variations across jurisdictions, the data indicated that HHR ratios across the United States increased more rapidly than overall population growth in 49 of 51 jurisdictions (96.1%). In contrast, in Canada, the increase in HHR ratios surpassed population growth in only 7 of 10 jurisdictions (70.0%). |
| 61 | Availability | - Supply analysis - Demand data (vacancies) | Powell JM, Kanny EM, Ciol MA.  State of the occupational therapy workforce: results of a national study.  Am J Occup Ther. 2008 Jan-Feb;62(1):97-105. | - This study determined the current status of the occupational therapy workforce in the United States with a demand-based report using current job data. - A 31-question survey was sent to rehabilitation administrators and managers from a proportional random sample of 556 facilities that hire occupational therapy practitioners in 29 states. Data were collected from November 2005 to February 2006 using structured mailing and follow-up procedures. - The response rate was 55%. | - The vacancy rate was 8.9% for occupational therapists and 7.7% for occupational therapy assistants. - Forty-five percent of respondents predicted an increase in occupational therapy positions in the next 2 years, and 30% predicted an increase in occupational therapy assistant positions. - Sixty-seven percent reported difficulty hiring occupational therapists, and 62% reported difficulty hiring occupational therapy assistants. |
| 62 | Availability | - Stocktaking and forecasting analysis of PT (US) - Influx and efflux variables are considered. - Use of demand indicators in addition to supply data to determine any shortages. | Landry MD, Hack LM, Coulson E, Freburger J, Johnson MP, Katz R, Kerwin J, Smith MH, Wessman HC, Venskus DG, Sinnott PL, Goldstein M.  Workforce Projections 2010-2020: Annual Supply and Demand Forecasting Models for Physical Therapists Across the United States.  Phys Ther. 2016, Vol. 96(1), pp. 71-80 | - The purpose of this study was to develop a strategy for modeling future workforce projections to serve as a basis for analyzing annual supply of and demand for physical therapists across the United States into 2020. - A traditional stock-and-flow methodology or model was developed and populated with publicly available data to produce estimates of supply and demand for physical therapists by 2020. - Supply was determined by adding the estimated number of physical therapists and the approximation of new graduates to the number of physical therapists who immigrated, minus US graduates who never passed the licensure examination, and an estimated attrition rate in any given year. - Demand was determined by using projected US population with health care insurance multiplied by a demand ratio in any given year. | - Three separate projection models were developed based on best available data in the years 2011, 2012, and 2013, respectively. - Based on these projections, demand for physical therapists in the United States outstrips supply under most assumptions. - Varying levels of attrition rates or permanent exits out of the profession can have important supply-side effects and appear to have an effect on predicting future shortage or surplus of physical therapists. |
| 63 | Availability | - Demand estimation in Beijing, China. | Bo W, Hong D, Xuezong L, Zhongxin X.  The demand for rehabilitation therapists in Beijing health organizations over the next five years.  Disabil Rehabil. 2008;30(5):375-80. | - To estimate the demand for rehabilitation therapists in Beijing health organizations over the next 5 years. - A literature search was carried out to confirm the distribution of rehabilitation therapists in Beijing health organizations in the past and to predict the demand for rehabilitation therapists in Beijing hospitals over the next 5 years by linear regression and logistic curve fitting. | - The total demand for rehabilitation therapists in Beijing in 2007 and 2010 will be 1480-1620 and 2240-2530, respectively. - The allocation and training of manpower specialized in rehabilitation therapeutics for the rehabilitation departments of polyclinics in Beijing should be improved. |
| 64 | Availability, Acceptability | - Cultural aspects about disability and rehabilitation in low-income countries. | Parnes, P Cameron D, Christie N, Cockburn L, Hasemi G, Yoshida K.  Disability in low-income countries: issues and implications.  Disabil Rehabil. 2009, Vol. 31(14), pp. 1170–1180. | - To critically examine the broad literature in the area of disability and development - Most of the data were collected from existing literature in the academic and practice settings and from the publications of key NGOs and governments. - It examines disability in the context of low-income countries, and then discusses key critical issues: disability and poverty, disability and health, disability and education, disability and gender, disability and children/youth, disability and conflict/natural disasters and disability and human rights. | - In all the areas analyzed, there were found reports of discrimination, stigmatisation and marginalisation. - Authors conclude that, as we address issues of multi-cultural disability services in developed countries, it is important to bear in mind the various issues that many people with disabilities and their families bring with them as the result of immigrating from a developing country. |
| 65 | Availability, Accessibility Acceptability | - Supply of rehabilitation providers. - Rural and remote regions - Cultural factors | Rathore FA, New PW, Iftikhar A.  A report on disability and rehabilitation medicine in Pakistan: past, present, and future directions.  Arch Phys Med Rehabil. 2011, Vol. 92(1), pp. 161-6. | - To present an overview of Physical Medicine and Rehabilitation (PM&R) in Pakistan, covering its origins, current status, and future directions. - An electronic literature search (1950-2009) was conducted using the Medline, Science Direct, Springer Link, CINAHL, and Google Scholar databases. - Statistical data were obtained from the Federal Bureau of Statistics. Interviews with pioneers of rehabilitation medicine in Pakistan, PWDs, and their families were conducted. | - There are only a few rehabilitation departments, and none have all the standard components of a rehabilitation team. - The number of practicing rehabilitation consultants is 38. There are an estimated 1000 physical therapists and 150 occupational therapists. - There is a need to increase the number of rehabilitation facilities significantly, staff them appropriately, and make them accessible to all who need them, including rural and remote regions where they are scarcer. - Discrimination is sometimes reported should be addressed by education and legislation. |
| 66 | Availability | - Influx from international migration (countries of origin). - Factors underlying international migration | Cornwall M, Keehn M, Lane M.  Characteristics of US-Licensed Foreign-Educated Physical Therapists.  Phys Ther. 2016, Vol. 96(3), pp. 293-304. | - To describe those physical therapists who are licensed in the United States but who were educated in another country. This description includes their country of education, their employment patterns, and the reasons they decided to emigrate and work as a physical therapist in the United States. - A cross-sectional survey was conducted. - An electronic survey was sent to all physical therapists currently licensed in the United States who had been educated in another country. Those who had been licensed within the last 5 years are reported. | - The results of the survey indicated that the typical foreign-educated physical therapist is female, aged 32.2 years, and was born and trained in either the Philippines or India. - The most common reasons cited as to why a particular jurisdiction was chosen for initial employment were "recruiter recommendation," "family, spouse, partner, or friends," "ease of the licensure process," and "ability to secure a visa sponsor." - A majority of foreign-educated physical therapists in this study initially worked in a skilled nursing facility, a long-term care or extended care facility, or a home health setting. |
| 67 | Availability | - Skill mix - Inter-professional competencies - Community-based rehabilitation | Kendall E, Muenchberger H, Catalano T, Amsters D, Dorsett P, Cox R.  Developing core interprofessional competencies for community rehabilitation practitioners: findings from an Australian study.  J Interprof Care. 2011 Mar;25(2):145-51. | - To determine the core competencies thatunderpin the practice of community rehabilitation (CR) practitioners working in a single state in Australia. - Using a recursive and consultative methodology designed to build consensus, CR professionals, trainers, educators, and researchers developed a preliminary set of core interprofessional competencies that were considered essential to their practice. | - Ten competencies emerged from this process. - Although there are limitations to the application of competencies, they will have significant implications for the future training of CR practitioners who can transcend professional boundaries. |
| 68 | Availability, Accessibility | - Community-bases rehabilitation workers. | Mannan H, Boostrom C, Maclachlan M, McAuliffe E, Khasnabis C, Gupta N.  A systematic review of the effectiveness of alternative cadres in community based rehabilitation.  Hum Resour Health. 2012, Vol. 10, p. 20. | - Systematic review to evaluate existing evidence regarding the effectiveness of alternative cadres working in CBR in low and middle income countries. - PUBMED, LILACS, SCIE, ISMEAR, WHOLIS, AFRICAN MED IND were searched, such as the online archive of the Asia Pacific Disability Rehabilitation Journal (available from 2002 to 2010), which was not covered by any of the other databases. There was no limit set on inclusion with regard to how recent a publication was in the general search. | - The search yielded 235 abstracts, only 6 of which addressed CBR through some type of evaluative component. Three of the studies explored the effects of CBR interventions, mainly related to physical disabilities, while three explored issues concerned with the work performance of rehabilitation workers. Altogether the studies covered four different countries. - All six studies related to specific service delivery in local contexts, using outcome measures that were not comparable across studies. We do not, therefore, feel that the current results provide adequate methodology or evidence for reliably generalizing their results. Due to the dearth of evidence regarding the effectiveness of alternative cadres in CBR, systematic research is needed on the training, performance and impacts of rehabilitation workers, including their capability of working across sectors and engaging with and making use of health systems research. |
| 69 | Accessibility | - Rural and remote practice (Australia) - Recruitment and retention - Models care delivery | Dew A, Bulkeley K, Veitch C, Bundy A, Gallego G, Lincoln M, Brentnall J, Griffiths S.  Addressing the barriers to accessing therapy services in rural and remote areas.  Disabil Rehabil. 2013, Vol. 35(18), pp. 1564-70. | - Throughout the world, people with a disability who live in rural and remote areas experience difficulty accessing a range of services including speech-, physio- and occupational therapy. This paper draws on information gathered from carers and adults with a disability living in a rural area in New South Wales (NSW), Australia to determine the extent to which people living in rural areas may receive a person-centred therapy service. - As part of a larger study, focus groups and individual interviews were conducted with 78 carers and 10 adults with a disability. Data were analysed using constant comparison and thematic analysis. | - Three related themes emerged: (i) travelling to access therapy; (ii) waiting a long time to get therapy; and (iii) limited access to therapy past early childhood. The themes overlaid the problems of recruiting and retaining sufficient therapists to work in rural areas. - Therapy service delivery in rural and remote areas requires: Place-based and person centred strategies to build local capacity in communities. Responsive outreach programs working with individuals and local communities. Recognition of the need to support families who must travel to access remotely located specialist services. Innovative use of technology to supplement and enhance service delivery. |
| 70 | Accessibility | - Rural and remote practice - Recruitment and retention | Roots RK, Li LC.  Recruitment and retention of occupational therapists and physiotherapists in rural regions: a meta-synthesis.  BMC Health Serv Res. 2013, Vol. 13, p. 59. | - This study aims to broaden the understanding of factors associated with recruitment and retention of OTs and PTs in rural regions, through a synthesis of evidence from qualitative studies found in the literature. - A systematic search of three databases was conducted for studies published between 1980 - 2009 specific to the recruitment and retention of OTs and PTs to rural areas. Studies deemed eligible were appraised using the McMaster Critical Review Form. - Employing an iterative process, a thematic analysis of studies and second order interpretations were made. | - Of the 615 articles retrieved, 12 qualitative studies met the eligibility criteria - Therapists' decision to locate, stay or leave rural communities was influenced to a greater degree by the availability of and access to practice supports, opportunities for professional growth and understanding the context of rural practice, than by location. - The second-order analysis revealed the benefits of a strength-based inquiry in determining recruitment and retention factors. - The themes that emerged were 1) support from the organization influences retention, 2) with support, challenges can become rewards and assets, and 3) an understanding of the challenges associated with rural practice prior to arrival influences retention. |
| 71 | Accessibility | - Distribution of Physical Therapists by region (Brazil) - Distribution of Physical Therapists by sector - Distribution of Physical Therapists by service Level | Costa LR, Costa JL, Oishi J, Driusso P.  Distribution of physical therapists working on public and private establishments in different levels of complexity of health care in Brazil.  Rev Bras Fisioter. 2012 Sep-Oct;16(5):422-30. | - To investigate the distribution of physical therapists in the levels of complexity of health care and between public and private establishments in Brazil. - A descriptive cross-sectional study was performed considering National Register of Health Service Providers-national bank data collected in March 2010 and demographic census 2010 data. | - Concentration of physical therapists in the specialized care, mostly in metropolis and big cities and in the private sector, with restricted to participation in the primary care. - The specialized establishments of private sector linked to the southeast region were the main sites of physical therapists. - 60% of physical therapists were linked to the private sector. Only 13% of all entries were linked to primary care. |
| 72 | Accessibility | - Rural/Remote Practice - Recruitment - Retention - Education | Winn C, Chisholm B, Hummelbrunner J  Factors affecting recruitment and retention of rehabilitation professionals in Northern Ontario, Canada: a cross-sectional study.  Rural Remote Health.2014;14:2619 | - Examining the factors that lead to rehabilitation professionals relocating to and remaining in the Northern Ontario. - Cross-sectional survey of rehabilitation professionals living and working in Northern Ontario administered in 2009. A total of 345 individuals completed the survey (response rate 57%). - Information included demographics and a rating of the personal and professional factors that had an impact on an individual's decision to continue living and working in the region. | - Differences were noted between those individuals originally from Northern Ontario and those who were not. - Rural or remote education experiences and rural/remote origin were identified as important recruitment factors. - Job satisfaction and lifestyle options were important factors for retention. |
| 73 | Accessibility | - Rural and Remote Practice - Recruitment and Retention - Rural and remote education programs | Winn CS, Chisholm BA, Hummelbrunner JA, Tryssenaar J, Kandler LS  Impact of the Northern Studies Stream and Rehabilitation Studies programs on recruitment and retention to rural and remote practice: 2002-2010.  Rural Remote Health. 2015 Apr-Jun;15(2):3126 | - Determining the number of NSS or RS program graduates who choose to live and work in Northern Ontario or other rural/remote areas, and the extent to which participation in these programs or other factors contributed to their decision. - Internet-based survey send to 83,6% of the eligible participants in 2011. The response rate was 52%. - 48 questions, including actors affecting recruitment and retention decisions. | - 33.9% reported having chosen rural or remote practice following graduation. - Individuals raised in a rural or remote community were 3.3 times more likely to work in a rural or remote community after graduation. - Recruitment was strongly associated with length of time immersed in rural/remote education settings and to participation in the NSS academic semester. - Job satisfaction, professional networking opportunities, and rural lifestyle options were identified as important factors for retention. |
| 74 | Accessibility | - Recruitment and retention strategies - Literature review and Expert panels | Tran D, Hall LM, Davis A, Landry MD, Burnett D, Berg K, Jaglal S.  Identification of recruitment and retention strategies for rehabilitation professionals in Ontario, Canada: results from expert panels.  BMC Health Serv Res. 2008 9;8:249. | - 1) to identify recruitment and retention strategies of rehabilitation professionals including occupational therapists, physical therapists and speech language pathologists from the literature; and 2) to investigate both the importance and feasibility of the identified strategies using expert panels amongst HHR and education experts. - A review of the literature was conducted to identify recruitment and retention strategies for rehabilitation professionals. Two expert panels, one on Recruitment and Retention and the other on Education were convened to determine the importance and feasibility of the strategies. A modified-delphi process was used to gain consensus and to rate the identified strategies along these two dimensions. | - 34 strategies were identified by the expert panels as being important and feasible for the development of a HHR plan for recruitment and retention of rehabilitation professionals. - 7 were categorized under the Quality of Worklife and Work Environment theme (e.g. providing assistance to rehabilitation professionals who work alone, particularly in rural environments) - 7 in Financial Incentives and Marketing (e.g. make professional development (PD) a regular part of budget planning), - 2 in Workload and Skill Mix (i.e. Implement a computerized database system with accessible real-time information to facilitate caseload management, (Use support personnel to increase efficiency of utilization of scarcer and higher order rehabilitation competencies (i.e. physiotherapy assistants or exercise therapists) - 13 in Professional Development (e.g. ensure access to professional colleagues/mentors for newly graduated practitioners through use of innovative communications technology) - 5 in Education and Training (e.g. develop and implement innovative programs that deliver continuing education to rehabilitative health care personnel located in remote locations) |
| 75 | Accessibility | - Rural versus urban: practice patterns. - Distribution of physical therapists. | Bath B, Gabrush J, Fritzler R, Dickson N, Bisaro D, Bryan K, Shah TI.  Mapping the Physiotherapy Profession in Saskatchewan: Examining Rural versus Urban Practice Patterns.  Physiother Can. 2015 Aug;67(3):221-31. | - 1) to compare demographic characteristics, professional engagement indicators, and clinical characteristics between physiotherapists practising in rural settings and those practising in urban settings and (2) to map the distribution of physiotherapists in Saskatchewan. - Cross-sectional study: de-identified data collected from the 2013 Saskatchewan College of Physical Therapists membership renewal (n=643), linked with the Saskatchewan Physiotherapy Association's (SPA) 2012 membership list and a list of physiotherapists who had served as clinical instructors. Employment location (rural vs. urban) was determined by postal code. | - Only 11.2% of Saskatchewan physiotherapists listed a rural primary employment location, and a higher density of physiotherapists per 10,000 people work in health regions with large urban centres. - Compared with urban physiotherapists, rural physiotherapists are more likely to provide direct patient care, to provide care to people of all ages, and to have a mixed client level. |
| 76 | Accessibility | - Rural and remote care (Australia) - Retention and recruitment (factors) | Gallego G, Dew A, Lincoln M, Bundy A, Chedid RJ, Bulkeley K, Brentnall J, Veitch C.  Should I stay or should I go? Exploring the job preferences of allied health professionals working with people with disability in rural Australia.  Hum Resour Health. 2015, Vol. 13, p. 5 | - This study aimed to determine the relative importance that allied health professionals AHPs (physiotherapists, occupational therapists, speech pathologists and psychologists - "therapists") living in a rural area of Australia and working with people with disability, place on different job characteristics and how these may affect their retention. - Cross-sectional survey was conducted using an online questionnaire distributed to AHPs working with people with disability in a rural area of Australia over a 3-month period. Information was sought about various aspects of the AHPs' current job, and their workforce preferences were explored using a best-worst scaling discrete choice experiment (BWSDCE). Conditional logistic and latent class regression models were used to determine AHPs' relative preferences for six different job attributes. - One hundred ninety-nine AHPs completed the survey; response rate was 51 %. | - "High autonomy of practice" is the most valued attribute level, followed by "travel BWSDCE arrangements: one or less nights away per month", "travel arrangements: two or three nights away per month" and "adequate access to professional development". - The least valued attribute levels were "travel arrangements: four or more nights per month", "limited autonomy of practice" and "minimal access to professional development". - Except for "some job flexibility", all other attributes had a statistical influence on AHPs' job preference. Preferences differed according to age, marital status and having dependent children. - This information can improve job designs in rural areas to increase retention in the underserved rural and remote regions of Australia. |
| 77 | Accessibility | - Recruitment and retention strategies - Models of service provision in underserved contexts (rural / remote areas) | Veitch C, Dew A, Bulkeley K, Lincoln M, Bundy A, Gallego G, Griffiths S.  Issues affecting therapist workforce and service delivery in the disability sector in rural and remote New South Wales, Australia: perspectives of policy-makers, managers and senior therapists.  Rural Remote Health. 2012;12:1903. | - To investigate issues of importance to policy-makers, managers and therapists providing services to people with disabilities in rural and remote areas of New South Wales (NSW), Australia. - Semi-structured interviews with individuals and small groups. - Participants included 12 policy-makers, 28 managers and 10 senior therapists from NSW government agencies and non-government organisations (NGOs) involved in providing related services. - Information was synthesised prior to using constant comparative analysis within and across data sets. | - Policy-related information transfer between organisations and employees was inconsistent. - Relatively recent innovations such as therapy assistants, information technology, and trans-disciplinary approaches, were raised as important service delivery considerations within the region. These and other innovations were expected to extend the coverage provided by therapists. - Participants recognised the need for therapists working for different organisations, in rural areas, to collaborate both in terms of peer support and service delivery to clients. - These elements may help people with disabilities in rural and remote areas of Australia to have access to appropriate rehabilitation care. |
| 78 | Access | - Rural recruitment and retention (factors) - Literature review. - Undergraduate rural programs | McAuliffe T, Barnett F.  Factors influencing occupational therapy students' perceptions of rural and remote practice.  Rural Remote Health. 2009 Jan-Mar;9(1):1078. | - To add to existing information about allied health students, particularly occupational therapy students, and rural and remote practice - To review the literature on occupational therapy students' perceptions of rural and remote practice. | - The shortage of rural and remote health practitioners is well documented. - Because rural and remote practice is characterised by a diversity of healthcare needs, rural health professionals need a variety of knowledge and skills. - A student's rural background was reported to be one of the strongest factors in their decision to work rurally, and an undergraduate rural program is one useful strategy to overcome the rural health professional shortage. - Undergraduate rural programs promote students' positive perceptions of rural and remote practice by exposure to a rural location, and factors such as rural fieldwork experience and fieldwork supervisors are likely to be influential. - Negative influential factors include a student's desire to work as a 'specialist', and personal, social and professional factors, such as a lack of professional development opportunities in a rural setting. |
| 79 | Accessibility | - Rural and remote areas (access to care) - Care Rationing - Retention | Adams R, Jones A, Lefmann S, Sheppard L.  Rationing is a reality in rural physiotherapy: a qualitative exploration of service level decision-making.  BMC Health Serv Res. 2015, Vol. 15, p. 121. | - Where demand exceeds available resources, decisions about resource allocation are required. - Qualitative approach enabled the researchers to explore participant perspectives about decisions informing rural physiotherapy service provision. - Stakeholder perspectives were obtained through surveys and in-depth interviews. - A system theory-case study heuristic provided a framework for exploration across sites within the investigation area: a large area of one Australian state with a mix of rural, regional and remote communities. - Thirty-nine surveys were received from participants in eleven communities. Nineteen in-depth interviews were conducted with physiotherapist and key decision-makers. | - Rationing of physiotherapy services was common to all sites of this study. - Rationing of services, more commonly expressed as service prioritisation, was more evident in responses of public sector physiotherapy participants compared to private physiotherapists. - However, private physiotherapists in rural areas reported capacity limits, including expertise, space and affordability that constrained service provision. - Increasing demand, organisational priorities, fiscal austerity measures and workforce challenges were identified as factors influencing both decision-making and service provision. - Decreased community access to local physiotherapy services and increased workforce stress, a key determinant of retention, are two results of such choices or decisions. |
| 80 | Accessibility | - Rural/Remote Practice - Scholarships - Support Systems | Devine SG, Williams G, Nielsen  Rural Allied Health Scholarships: do they make a difference?  Rural Remote Health. 2013 Oct-Dec;13(4):2459. | - To analyze the impact of scholarships on workforce outcomes particularly in relation to allied health professionals. - Mixed methods study involving quantitative analysis of existing Queensland Health scholarship data and a qualitative study that used one-on-one, in-depth telephone interviews with 17 past or current scholarship holders and 11 managers of scholarship holders. | - Key motivators for applying for a scholarship were financial and job security upon graduation, although the general appeal of and preference for rural practice was an underlying motivator. - Regardless of receiving a scholarship, most scholarship recipients reported they would have gone into rural and remote practice. - New graduates need extra support in transition to practice, while health services shall have well defined, consistent, operational processes that orient new graduates, particularly in relation to supervision, mentoring and professional development. |
| 81 | Accessibility | - Innovative model of rehabilitation delivery in underserved locations(enhancing access). - Tele-rehabilitation | Lincoln M, Hines M, Fairweather C, Ramsden R, Martinovich J.  Multiple stakeholder perspectives on teletherapy delivery of speech pathology services in rural schools: a preliminary, qualitative investigation.  Int J Telerehabil. 2015, Vol. 6(2), pp. 65-74 | - To investigate stakeholders' views on the feasibility and acceptability of a pilot speech pathology teletherapy program for children attending schools in rural New South Wales, Australia. - Nine children received speech pathology sessions delivered via Adobe Connect® web-conferencing software. - During semi-structured interviews, school principals (n = 3), therapy facilitators (n = 7), and parents (n = 6) described factors that promoted or threatened the program's feasibility and acceptability. - Themes were categorized according to whether they related to (a) the use of technology; (b) the school-based nature of the program; or (c) the combination of using technology with a school-based program. | - Despite frequent reports of difficulties with technology, teletherapy delivery of speech pathology services in schools was highly acceptable to stakeholders. - The use of technology within a school environment increased the complexities of service delivery. - Service providers should pay careful attention to planning processes and lines of communication in order to promote efficiency and acceptability of teletherapy programs. |
| 82 | Accessibility, Quality | - Prosthetic and orthotic services in a underserved country. - Conditions for providing quality of care | Magnusson L, Ahlström G.  Experiences of providing prosthetic and orthotic services in Sierra Leone--the local staff's perspective.  Disabil Rehabil. 2012, Vol. 34(24), pp. 2111-8. | - The aim of this qualitative study was to explore the experiences of prosthetic and orthotic service delivery in Sierra Leone from the local staff's perspective. - Fifteen prosthetic and orthotic technicians working at all the rehabilitation centres providing prosthetic and orthotic services in Sierra Leone were interviewed. The interviews were transcribed and subjected to latent content analysis. | - One main theme emerged: sense of inability to deliver high-quality prosthetic and orthotic services. - This main theme was generated from eight sub-themes: Desire for professional development; appraisals of work satisfaction and norms; patients neglected by family; limited access to the prosthetic and orthotic services available; problems with materials and machines; low public awareness concerning disabilities; marginalisation in society and low priority on the part of government. - Support from international organisations was considered necessary as well as educating more prosthetic and orthotic staff to a higher level. |
| 83 | Accessibility | - Barriers for accessing care in African countries. | Eide AH, Mannan H, Khogali M, van Rooy G, Swartz L, Munthali A, Hem KG, MacLachlan M, Dyrstad K.  Perceived Barriers for Accessing Health Services among Individuals with Disability in Four African Countries.  PLoS One. 2015, Vol. 10(5), p. e0125915. | - The current study aimed at identifying the magnitude of specific barriers, and to estimate the impact of disability on barriers for accessing health care in general. - A population based household survey was carried out in Sudan, Namibia, Malawi, and South Africa, including a total of 9307 individuals. - The sampling strategy was a two-stage cluster sampling within selected geographical areas in each country. - A listing procedure to identify households with disabled members using the Washington Group six screening question was followed by administering household questionnaires in households with and without disabled members, and questionnaires for individuals with and without disability. | - Lack of transport, availability of services, inadequate drugs or equipment, and costs, are the four major barriers for access. - The study also showed substantial variation in perceived barriers, reflecting largely socio-economic differences between the participating countries. - Urbanity, socio-economic status, and severity of activity limitations are important predictors for barriers. |
| 84 | Accessibility | - Barriers for accessing care in rural Namibia | Van Rooya G, Amadhilaa E, Mufuneb P, Swartzc L, Mannand H, MacLachland M.  Perceived barriers to accessing health services among people with disabilities in rural northern Namibia.  Disability & Society. 2012, Vol. 27, pp. 761-775 | - People living with disabilities (PWD) face unique problems in dealing with conventional healthcare facilities. - The study investigates the experiences of PWD as they access healthcare facilities in rural Namibia. - More specifically, it investigates structural–environmental and process barriers to accessing health facilities. - The study relied on semi-structured interviews and purposive sampling. | - PWD find it difficult to walk to health centers for treatment due to lack of transport, money to pay for treatment and toilet facilities and the distance is too far for people with lower-limb disabilities. - There is a need to consider the unique issues affecting access to healthcare for people living with disabilities to achieve equitable access to healthcare services. |
| 85 | Accessibility | - Barriers for accessing care in a low-income area | Maart S, Jelsma J.  Disability and access to health care – a community based descriptive study.  Disabil Rehabil. 2014, Vol. 36, pp. 1489-93. | - Participants in a large community based survey in a low-income area were asked questions relating to their use of health related services. - Using random, cluster sampling a representative sample of 1083 households in a deprived area of Cape Town were approached and 152 people with disability were interviewed. | - The percentages reporting unmet needs were respectively: 54% for home-based care; 34.5% for assistive devices, 28.9% for medical rehabilitation services; and 2.5% for health services. - Those over 65 years of age were less likely to have had the medical rehabilitation that they required (χ² = 8.00, p = 0.018). - There were fewer respondents with sensory and language disorders but these groups reported proportionately more unmet needs. - The main problems with accessing services included inadequate finances (71%) and transport problems (72%). |
| 86 | Accessibility | - Barriers for accessing care in South India | Gudlavalleti MV, John N, Allagh K, Sagar J, Kamalakannan S, Ramachandra SS; South India Disability Evidence Study Group.    Access to health care and employment status of people with disabilities in South India, the SIDE (South India Disability Evidence) study.  BMC Public Health. 2014 Nov 1;14:1125. | - The study was conducted in one district each in two States (Andhra Pradesh and Karnataka) in 2012. - Appropriate age and sex-matched people without a disability were recruited to compare with people with disability who were identified through a population-based survey and available government disability records by trained key informants. - Investigators administered questionnaire schedules to people with and without a disability to harness information on employment and health service access, utilization and barriers. - A total of 839 people with disabilities and 1153 age and sex matched people without a disability, aged 18 years or more were included. | - People with disability faced significantly more barriers to accessing health services compared to people without a disability. - Barriers included ignorance regarding availability of services, costs of services and transportation. - This study highlights the challenges that people with disability face in accessing health-care and employment opportunities. - The study findings have public health implications and should be used for planning need-based appropriate strategies to improve health care access for people with disabilities. |
| 87 | Accessibility Acceptability, Quality | - Barriers to rehabilitation access (rural Kenya) - Profession-focused service delivery. - Inconsistent recording practices. | Bunning K, Gona JK, Odera-Mung'ala V, Newton CR, Geere JA, Hong CS, Hartley S.  Survey of rehabilitation support for children 0-15 years in a rural part of Kenya.  Disabil Rehabil. 2014, Vol. 36(12), pp. 1033-41. | - To establish the scope and nature of rehabilitation support available to children with disabilities (0-15 years) and their families in rural Kenya. - A comprehensive sample comprising service provision in the health and special education sectors was established. Non-governmental and community-based organisations were also included. - A survey of rehabilitation services was conducted through examination of service-related documentation and key informant interviews with the heads of services. | - Provision was challenged by inadequate staffing, resources and transport. Government funding was supplemented variously by donations and self-sufficiency initiatives. - Rehabilitation approaches appeared to be informed by professional background of practitioner, rather than the needs of child. - Service documentation revealed use of inconsistent recording methods. - Better skills and mechanisms for recording, monitoring and evaluating practice are needed. |
| 88 | Accessibility | - Barriers to rehabilitation access in rural communities. - Community- and home-based option for enhancing access | Gona JK, Newton CR, Geere JA, Hartley S.  Users' experiences of physiotherapy treatment in a semi-urban public hospital in Kenya.  Rural Remote Health. 2013, Vol. 13(3), p. 2210 | - This study explores users' perceptions of physiotherapy, challenges faced by users, possible options for management, and determines whether current physiotherapy practice in a rural Kilifi District general hospital in Kenya facilitates future self-management of chronic conditions. - Eight in-depth interviews, 3 focus groups discussions and 4 participant observations were conducted for data collection. - Inductive data analysis was used to generate themes from the rich-text data of the transcriptions. | - Challenges included distance from health facilities, negative experiences with some therapists, and lack of staff and equipment. - Rehabilitation options included community- and home-based programs fostering self-management of chronic conditions. - Current hospital practice lacks emphasis on self-management skills for patients with chronic conditions who can do their physiotherapy at home. - There is therefore need for community-based services that place emphasis on self-management of chronic conditions for fostering better health outcomes in rural communities. |
| 89 | Accessibility | - Enhancing access to rehabilitation by a home-based rehabilitation program. | Cobbing S, Hanass-Hancock J, Myezwa H.  A home-based rehabilitation intervention for people living with HIV and disability in a resource-poor community, KwaZulu-Natal: study protocol for a randomised controlled trial.  Trials. 16, 2015, p. 491 | - There is a dearth of evidence, however, related to home-based rehabilitation interventions for people living with HIV, particularly in sub-Saharan Africa - the region with the highest global prevalence of HIV. - A randomised controlled trial design will be employed. - Individuals randomly allocated to the intervention group will participate in a four-month home-based rehabilitation programme, conducted once a week in their homes. - This programme will be implemented by community workers who will be trained and supervised by a qualified physiotherapist. | - The researchers aim to employ a novel task shifting approach to implement a needs-based home-based rehabilitation programme for people living with HIV in order to improve their quality of life and functional ability. - It is hoped that this study will provide rehabilitation professionals and researchers with evidence that can be utilised to improve existing rehabilitation interventions for people living with HIV. |
| 90 | Accessibility | - Community-based rehabilitation as a mean to enhance access to rehabilitation care | Cleaver S, Nixon S.  A scoping review of 10 years of published literature on community-based rehabilitation.  Disabil Rehabil. 2014, Vol. 36(17), pp. 1385-94 | - To identify the characteristics of peer-reviewed literature on community-based rehabilitation (CBR) in low- and middle-income countries published in English from 2003 to 2012. - Systematic search of electronic databases using specific keyword/subject heading combinations. - Journal articles were included if they were published in English, used "CBR" as related to rehabilitation with persons with disabilities and not limited to high-income countries (HICs). - Data were charted according to both pre-determined and emergent categories. A subset of articles was charted by two reviewers to ensure reliability of variables. | - A total of 114 articles were included. Fifty-two articles presented empirical research and 49 were published in one of two journals. The articles represented CBR activity in 26 specific countries, although only two of these were in Europe and only one was in the Americas. - Authors were predominantly affiliated at universities and in HICs. - Community-based rehabilitation (CBR) has been promoted as a rehabilitation strategy of choice in low- and middle-income countries (LMICs), but it has been critiqued for lack of an evidence base. - A large number (114) of peer-reviewed articles were published on CBR between 2003 and 2012. - Just under half of these articles (45%) presented empirical research, indicating that the evidence base for CBR is growing but will benefit from continued, rigorous inquiry. R - esearchers from LMICs appear to be largely under-represented in published CBR research, flagging the need to support LMIC partners to share their CBR research in peer-reviewed journals. |
| 91 | Accessibility, Quality | - Tele-rehabilitation as a mean to enhance access to rehabilitation - Cost-effectiveness | Kairy D, Lehoux P, Vincent C, Visintin M.  A systematic review of clinical outcomes, clinical process, healthcare utilization and costs associated with telerehabilitation.  Disabil Rehabil. 2009, Vol. 31(6), pp. 427-47. | - To identify clinical outcomes, clinical process, healthcare utilization and costs associated with telerehabilitation for individuals with physical disabilities. - Relevant databases were searched for articles on telerehabilitation published until February 2007. Reference lists were examined and key journals were hand searched. - Studies that included telerehabilitation for individuals with physical impairments and used experimental or observational study designs were included in the analysis, regardless of the specific clientele or location of services. - Data was extracted using a form to record methodological aspects and results relating to clinical, process, healthcare utilization and cost outcomes. - Study quality of randomized clinical trials was assessed using the PEDro rating scale. | - Some 28 articles were analysed. These dealt with rehabilitation of individuals in the community, neurological rehabilitation, cardiac rehabilitation, follow-up of individuals with spinal cord injuries, rehabilitation for speech-language impairments, and rehabilitation for varied clienteles. - Clinical outcomes were generally improved following a telerehabilitation intervention and were at least similar to or better than an alternative intervention. - Clinical process outcomes, such as attendance and compliance, were high with telerehabilitation although few comparisons are made to alternative interventions. - Consultation time tended to be longer with telerehabilitation. Satisfaction with telerehabilitation was consistently high, although it was higher for patients than therapists. - Only five of the studies examined costs. There is some preliminary evidence of potential cost savings for the healthcare facility. |
| 92 | Accessibility | - Direct access to PT as a mean to enhance access to rehabilitation | Bury TJ, Stokes EK.  A global view of direct access and patient self-referral to physical therapy: implications for the profession.  Phys Ther. 2013, Vol. 93(4), pp. 449-59 | - The purpose of this study was to map the presence of direct access to physical therapy services in the member organizations of the World Confederation for Physical Therapy (WCPT) in the context of physical therapist practice and health systems. - A 2-stage, mixed-method, descriptive study was conducted. - A purposive sample of member organizations of WCPT in Europe was used to refine the survey instrument, followed by an online survey sent to all WCPT member organizations. - Data were analyzed using descriptive statistics, and content analysis was used to analyze open-ended responses to identify themes. - A response rate of 68% (72/106) was achieved. | - Direct access to physical therapy was reported by 58% of the respondents, with greater prevalence in private settings. - Organizations reported that professional (entry-level) education equipped physical therapists for direct access in 69% of the countries. - National physical therapy associations (89%) and the public (84%) were thought to be in support of direct access, with less support perceived from policy makers (35%) and physicians (16%). - Physical therapists' ability to assess, diagnose, and refer patients on to specialists was more prevalent in the presence of direct access. - Professional legislation, the medical profession, politicians, and policy makers are perceived to act as both barriers to and facilitators of direct access. |
| 93 | Accessibility | - Direct Access to PT | Ojha HA, Snyder RS, Davenport TE.  Direct access compared with referred physical therapy episodes of care: a systematic review.  Phys Ther. 2014, Vol. 94(1), pp. 14-30 | - To conduct a systematic review of the literature on patients with musculoskeletal injuries and compare health care costs and patient outcomes in episodes of physical therapy by direct access compared with referred physical therapy. - Relevant databases were searched using terms related to physical therapy and direct access. Included articles were hand searched for additional references. - Included studies compared data from physical therapy by direct access with physical therapy by physician referral, studying cost, outcomes, or harm. The studies were appraised using the Centre for Evidence-Based Medicine (CEBM) levels of evidence criteria and assigned a methodological score. | - Of the 1,501 articles that were screened, 8 articles at levels 3 to 4 on the CEBM scale were included. - There were statistically significant and clinically meaningful findings across studies that satisfaction and outcomes were superior, and numbers of physical therapy visits, imaging ordered, medications prescribed, and additional non-physical therapy appointments were less in cohorts receiving physical therapy by direct access compared with referred episodes of care. - There was no evidence for harm. - There is evidence across level 3 and 4 studies (grade B to C CEBM level of recommendation) that physical therapy by direct access compared with referred episodes of care is associated with improved patient outcomes and decreased costs. - Physical therapy by way of direct access may contain health care costs and promote high-quality health care. Third-party payers should consider paying for physical therapy by direct access to decrease health care costs and incentivize optimal patient outcomes. |
| 94 | Accessibility, Quality | - Direct Access to Physical Therapy. - Referral of patients to physicians. | Boissonnault WG, Ross MD.  Physical therapists referring patients to physicians: a review of case reports and series.  J Orthop Sports Phys Ther. 2012 May;42(5):446-54. | - To summarize published patient case reports that described physical therapist/patient episodes of care that resulted in the referral of the patient to a physician and a subsequent diagnosis of medical disease. - A literature search identified 78 case reports describing physical therapist referral of patients to physicians with subsequent diagnosis of a medical condition. Two evaluators reviewed the cases and summarized 5 different types of relevant information. | - This review of published patient case reports provides numerous examples of physical therapists using effective multifactorial screening strategies for referred and direct-access patients, leading to timely patient referrals to physicians. - The therapist-initiated patient referral to a physician led to subsequent diagnosis of a wide range of conditions and pathological processes. |
| 95 | Accessibility | - Disparities in access and use of rehabilitation services. | Freburger JK, Holmes GM, Ku LJ, Cutchin MP, Heatwole-Shank K, Edwards LJ.  Disparities in postacute rehabilitation care for stroke: an analysis of the state inpatient databases.  Arch Phys Med Rehabil. 2011, Vol. 92(8), pp. 1220-9 | - To determine the extent to which sociodemographic and geographic disparities exist in the use of postacute rehabilitation care (PARC) after stroke. - Cross-sectional analysis of data for 2 years (2005-2006) from the State Inpatient Databases. - All short-term acute-care hospitals in 4 demographically and geographically diverse states. - Participants: Individuals (age, ≥45y; mean age, 72.6y) with a primary diagnosis of stroke who survived their inpatient stay (N=187,188). The sample was 52.4% women, 79.5% white, 11.4% black, and 9.1% Hispanic. | - Multilevel logistic regression analyses were conducted to identify sociodemographic and geographic disparities in PARC use, controlling for illness severity/comorbid conditions, hospital characteristics, and PARC supply. - Several sociodemographic and geographic disparities in PARC use were identified. - Blacks, women, older individuals, and those with lower incomes were more likely to receive institutional care; Hispanics and the uninsured were less likely. - Racial minorities, women, older individuals, and those with lower incomes were more likely to receive HH care; uninsured individuals were less likely. - Blacks, women, older individuals, the uninsured, and those with lower incomes were more likely to receive SNF versus IRF care. - PARC use varied significantly by hospital and geographic location. |
| 96 | Accessibility | - Attractiveness (recruitment and retention). - Employment sectors | Landry MD, Hastie R, Oñate K, Gamble B, Deber RB, Verrier MC.  Attractiveness of employment sectors for physical therapists in Ontario, Canada (1999-2007): implication for the long term care sector.  BMC Health Serv Res. 2012 May 29;12:133. | - Use of the concepts of inflow and stickiness to assess the relative attractiveness of sectors for physical therapists (PTs) in Ontario, Canada. - A longitudinal dataset of registered PTs in Ontario (1999-2007) was created, and primary employment sector was categorized as 'hospital', 'community', 'long term care' (LTC) or 'other.' Inflow and stickiness values were then calculated for each sector, and trends were analyzed. | - Inflow grew across all sectors, but the LTC sector had the highest inflow of 32.0%. - PTs practicing in hospitals had the highest stickiness: 87.4%. - The community and other employment sectors had stickiness values of 78.2% and 86.8% respectively, while the LTC sector had the lowest stickiness of 73.4%. - Among all employment sectors, LTC had highest inflow but lowest stickiness. - Despite highest overall proportional growth or inflow, more PTs in LTC migrate out of the sector compared to any other sector, while the LTC sector is growing at a much faster rate than others. - The LTC sector is the least attractive employment sector across the care continuum in physical therapy. |
| 97 | Accessibility | - Recruitment and retention strategies. - Employment sectors | Tran D, Davis A, McGillis Hall L, Jaglal SB.  Comparing Recruitment and Retention Strategies for Rehabilitation Professionals among Hospital and Home Care Employers.  Physiother Can. 2012 Winter;64(1):31-41. | - To compare hospital and home care employers' rankings of both the importance and the feasibility of workforce strategies for recruiting and retaining rehabilitation professionals - An online self-administered questionnaire was distributed to all employers of rehabilitation professionals in Ontario hospitals (n=144) and Community Care Access Centre home care providers (n=34). The response rate was 50% from hospitals and 73.5% from home-care settings. - Importance and feasibility rankings were based on the percentage of high ratings; 95% CIs were used to determine significant differences between hospital and home care rankings. | - The strategies considered most important and feasible for rehabilitation therapists, regardless of setting, were: communication between employer and worker, compensation packages, access to research, and professional development in budget planning. - Tangible resources, support personnel, work safety, and marketing rehabilitation careers to high school students were ranked significantly higher by hospitals than by home care providers. - Strategies focusing on rural and remote areas, such as orientation packages, mentors, and minimizing isolation, are more important in home care settings than in hospitals. |
| 98 | Acceptability | - Cardiac rehabilitation program at an Aboriginal Medical Service | Dimer L, Dowling T, Jones J, Cheetham C, Thomas T, Smith J, McManus A, Maiorana AJ.  Build it and they will come: outcomes from a successful cardiac rehabilitation program at an Aboriginal Medical Service.  Aust Health Rev. 2013, Vol. 37(1), pp. 79-82 | - Cardiovascular disease (CVD) is the leading disease burden in Aboriginal Australians, but culturally appropriate cardiac rehabilitation programs are lacking. - The study evaluated the uptake and effects on lifestyle, and cardiovascular risk factors, of cardiac rehabilitation at an Aboriginal Medical Service (AMS). - The program involved weekly exercise and education sessions (through 'yarning') for Aboriginal people with or at risk of CVD. Participants' perceptions of the program and the impact on risk factors were evaluated following 8 weeks of attendance. | - AMS-based cardiac rehabilitation was well attended, and improved cardiovascular risk factors and health management. - An AMS is an ideal location for managing cardiovascular health and provides a setting conducive to addressing a broad range of chronic conditions |
| 99 | Acceptability | - Cardio-pulmonary rehabilitation for an Aboriginal population. | Davey M, Moore W, Walters J.  Tasmanian Aborigines step up to health: evaluation of a cardiopulmonary rehabilitation and secondary prevention program.  BMC Health Serv Res. 2014 Aug 18;14:349. | - This study evaluated the uptake and effectiveness of a cardiovascular and pulmonary rehabilitation program specifically designed and provided for the Aboriginal community, by the Tasmanian Aboriginal Centre, for people with diagnosed chronic heart or respiratory disease and those at high risk of developing such conditions. - Rehabilitation programs (n = 13) comprised two exercise and one education session per week over eight weeks. - Data, collected at baseline and on completion, included health status, risk factors, attendance, anthropometric measurements, physical capacity and quality of life. - Qualitative written feedback from participants and staff was analysed thematically. | - Participants lost weight, and waist circumference decreased (mean -3.6 cm, 95% confidence interval (CI)-2.5 to -4.7). There were clinically significant improvements in six-minute walk distance (mean 55.7 m, 95% CI 37.8 to 73.7) and incremental shuttle walk (mean 106.2 m, 95% CI 79.1 to 133.2). There were clinically significant improvements in generic quality of life domains, dyspnoea and fatigue. - Three factors that facilitated participation: support from peers and health workers, provision of transport and the program structure. - Participants' awareness of improvements in their health contributed to ongoing participation and positive health outcomes, and participants would recommend the program to family and friends. - A cardiopulmonary program, which included exercise and education and met national guidelines, was designed and delivered specifically for the Aboriginal community. - It increased participation in rehabilitation by Aborigines with, or at high risk of, established disease and led to positive changes in health behaviours, functional exercise capacity and health related quality of life. |
| 100 | Acceptability | - Development of a culturally-relevant intervention (in Kenya) | Hartley S, Murira G, Mwangoma M, Carter J, Newton CR.  Using community/researcher partnerships to develop a culturally relevant intervention for children with communication disabilities in Kenya.  Disabil Rehabil. 2009;31(6):490-9.. | - To develop a culturally relevant community-based intervention for children with communication disabilities in Kenya through a community/researcher partnership. - Using a qualitative approach, initial data was collected through focus group discussions with women, disabled people and traditional dancers. The groups examined the needs, problems and challenges faced by disabled children and their families. - This generated the content and structure for a series of participatory workshops with a further two women's groups. - These workshops strove to generate a culturally relevant community-based intervention programme for children with communication disabilities and their families. - The content of the resulting intervention was observed to be different from existing programmes described in the literature. - Notably it included many culturally appropriate strategies for increasing social integration and raising community awareness. | - The process of generating a locally relevant community-based rehabilitation intervention is potentially transferable and has particular relevance to the estimated 80% of the world where there are no formal rehabilitation services for children with disabilities and where women's groups are a strong element of local culture. - (i) Community/researcher partnerships can be used to develop interventions; (ii) such interventions are different from those imported from other cultures; and (iii) this process is transferable and can be part of the preparations for a Randomized Control Trial. |
| 101 | Acceptability | - Clinical education in low-income countries (from high-income) - Overcoming ethical and practical barriers | Ahluwalia P, Cameron D, Cockburn L, Ellwood L, Mori B, Nixon SA.  Analyzing international clinical education practices for Canadian rehabilitation students.  BMC Med Educ. 2014, Vol. 14, p. 187. | - Clinical training in low-income countries has become increasingly popular among pre-licensure trainees from high-income countries. - The Working Group on Ethics Guidelines for Global Health Training ("WEIGHT Guidelines") were designed to identify and inform the complex and contentious field of international clinical education. - The study aimed to use the WEIGHT Guidelines to evaluate an international clinical internship programme for Master's-level rehabilitation students at a Canadian university. - In-depth, semi-structured interviews were conducted with eight Canadian rehabilitation researchers, educations and/or clinicians responsible for administering international internships across three clinical training programmes. - Interview questions were informed by the WEIGHT Guidelines. Directed content analysis was used to identify priorities for policy, practice and research. | - Five themes relating to strengthening international clinical education were identified: (1) from one-time internships to long-term partnerships, (2) starting a discussion about "costs", (3) a more informed approach to student selection, (4) expanding and harmonizing pre-departure training across disciplines, and (5) investing in post-internship debriefing. - International clinical education is fraught with ethical, pedagogical and logistical issues that require recognition and ongoing management. - This is the first study to use the WEIGHT Guidelines as a qualitative research tool for assessing an existing global health education programme. - Results highlight new priorities for action at the Canadian "sending institution", including more explicit attention to the costs (broadly defined) borne by all parties. - A crucial next step is deepened engagement with educational partners at the "receiving organizations" based in low-income countries to nurture dialogue regarding reciprocity, trust and sustainability of the partnership. - Education research is also needed that evaluates models of pre-departure training and post-internship debriefing for trainees. |
| 102 | Accessibility | - Clinical education and service learning across countries within a global health perspective | Pechak C1, Thompson M  Going global in physical therapist education: International Service-Learning in US-based programmes.  Physiother Res Int. 2011 Dec;16(4):225-36.. | - Physical therapist education programmes increasingly offer international opportunities such as International Service-Learning (ISL) to their students. - A descriptive, exploratory study was performed using grounded theory. Snowball and purposive, theoretical sampling yielded 14 faculty members with experience in international service, international learning or ISL in physical therapist education programmes. - Faculty were interviewed by phone. Interview transcriptions and course documents were analysed applying grounded theory methodology. - Data from eight programmes which met the operational definition of established ISL were used to address the purposes of this paper. | - Five phases of establishing an ISL programme were identified: development, design, implementation, evaluation, and enhancement. Although no single model exists for ISL in physical therapist education; commonalities in structures and processes were identified in each phase. - While analysis revealed that each programme shared commonalities and demonstrated differences in structures and processes compared with the other programmes, the study demonstrated a general lack of focus on formal community outcomes which raises ethical concerns. - Future research and dialogue is warranted to explore ethics and good practice in ISL and other global health initiatives in physical therapy. |
| 103 | Acceptability | - Competencies for working in low-income locations | Cassady C, Meru R, Chan NM, Engelhardt J, Fraser M, Nixon S.  Physiotherapy beyond Our Borders: Investigating Ideal Competencies for Canadian Physiotherapists Working in Resource-Poor Countries.  Physiother Can. 2014, Vol. 66(1), pp. 15-23. | - To explore the perspectives of Canadian physiotherapists with global health experience on the ideal competencies for Canadian physiotherapists working in resource-poor countries. - A qualitative interpretive methodology was used, and the Essential Competency Profile for Physiotherapists in Canada, 2009 (ECP), was employed as a starting point for investigation and analysis. - Semi-structured one-on-one interviews (60-90 minutes) were conducted with 17 Canadian physiotherapists who have worked in resource-poor countries. - Descriptive and thematic analyses were conducted collaboratively. | - The seven ECP roles-Expert, Communicator, Collaborator, Manager, Advocate, Scholarly Practitioner, and Professional-were all viewed as important for Canadian physiotherapists working in resource-poor countries. - Two roles, Communicator and Manager, have additional competencies that participants felt were important. Communicator: Communication skills for differences in language and culture; Manager: Creativity and Resourcefulness. - Three novel roles-Global Health Learner, Critical Thinker, and Respectful Guest-were created to describe other competencies related to global health deemed crucial by participants. |
| 104 | Acceptability | - International clinical education: overcoming barriers | Crawford E, Biggar JM, Leggett A, Huang A, Mori B, Nixon SA, Landry MD.    Examining international clinical internships for canadian physical therapy students from 1997 to 2007.  Physiother Can. 2010, Vol. 62(3), pp. 261-73. | - To describe international clinical internships (ICIs) for Canadian physical therapy (PT) students, explore the experiences of individuals involved in ICIs, and develop recommendations for future ICIs based on these findings. - This study employed a mixed-methods approach. - An online questionnaire surveyed academic coordinators of clinical education (ACCEs, n=14) on the availability, destinations, and number of ICIs from 1997 to 2007. - Semi-structured telephone interviews were then conducted with eight PT students, seven ACCEs, and three supervising clinicians to investigate their ICI experiences. - Interview transcripts were coded descriptively and thematically using NVivo. | - ICIs are currently available at 12 of 14 Canadian PT schools. A total of 313 students participated in ICIs in 51 different destination countries from 1997 to 2007. - Over this period, increasing numbers of students participated in ICIs and developing countries represented an increasing proportion of ICI destinations. - Key themes identified in the interviews were opportunities, challenges, and facilitating factors. - Recommendations to enhance the quality of future ICIs are (1) clearly defined objectives for ICIs, (2) additional follow-up post-ICI, and (3) improved record keeping and sharing of information on ICI destination countries and host sites. |
| 105 | Acceptability | - International fieldwork: overcoming barriers | Cameron D, Cockburn L, Nixon S, Parnes P, Garcia L, Leotaud J, MacPherson K, Mashaka PA, Mlay R, Wango J, Williams T.  Global partnerships for international fieldwork in occupational therapy: reflection and innovation.  Occup Ther Int. 2013 Jun;20(2):88-96 | - International fieldwork placements (IFPs) have become very popular among healthcare students including those in occupational therapy programmes. There are many potential benefits that can accrue to the students; however, there are critiques of international placements especially for students going to underserviced areas. - The purpose of this paper is to provide a case study/model programme description that critically reflects on six partnerships in three underserviced countries that provide IFPs to students from one Canadian university. - The personal opinions of each partner were collected verbally, by email and by a qualitative review of the past 10 years of partnership interaction. - Direct input of students is not utilized, although feedback given to co-authors by students is reflected. | - Benefits include the development of an increased number of sustainable long-term quality placements, orientation materials, student supports and the involvement of university faculty in research and capacity building projects in partner countries. - Challenges include the need for an expanded formal agreement, more bilateral feedback and examination of supervision models. - More research is needed on perspectives of partners in IFPs, impact of IFPs on clinical practice in student's home countries, impact of IFPS on underserviced areas and effective strategies for debriefing. |
| 106 | Acceptability | - Cultural- compete training | Chipps JA, Simpson B, Brysiewicz P.  The effectiveness of cultural-competence training for health professionals in community-based rehabilitation: a systematic review of literature.  Worldviews Evid Based Nurs. 2008, Vol. 5(2), pp. 85-94 | - To find and review studies in which investigators evaluated cultural-competence training in community-based rehabilitation settings; critique study methods, describe clinical outcomes, and make recommendations for future research. - A review of the effectiveness of cultural-competence training for health professionals in community-based rehabilitation settings was conducted. - Research citations from 1991-2006 in CINAHL, Medline, Pubmed, PsycInfo, SABINET, Cochrane, Google, NEXUS, and unpublished abstracts were searched. - Searching, sifting, abstracting, and assessing quality of relevant studies by three reviewers. Studies were evaluated for sample, design, intervention, threats to validity, and outcomes. | - Five studies and one systematic review were evaluated. Positive outcomes were reported for most training programs. - Reviewed studies generally had small samples and poor design. - The paucity of studies and lack of empirical precision in evaluating effectiveness necessitate future studies that are methodologically rigorous to allow confident recommendations for practice. |
| 107 | Acceptability | - Cultural factors - Scope of practice | Wickford J, Hultberg J, Rosberg S.  Physiotherapy in Afghanistan--needs and challenges for development.  Disabil Rehabil. 2008, Vol. 30(4), pp. 305-13 | - To describe and analyse the current situation of the physiotherapy component of the Rehabilitation of Afghans with Disability (RAD) programme, in order to identify the needs and challenges for further development. - The study was conducted as a field study with an anthropological approach by means of participant observation, unstructured and semi-structured interviews and photography. | - The therapists in RAD work in isolation with little opportunity for further education or professional development. Their approach is mainly medical, where the work is dictated by the patients' expectations and doctors' recommendations. - They use primarily passive methods of treatment, and their work is affected by cultural, religious and situational factors. - There is a need for further development of physiotherapy in Afghanistan. Active and individually adapted treatment methods, clinical reasoning processes and evidence-based practice should be encouraged. - There are several challenges in this, based on Afghan culture and traditions, gender issues, religious factors, an authoritative society, a medical approach in treatment, and isolation and limitations in access to information. |
| 108 | Acceptability | - Cultural factors. - Gender issues | Magnusson L, Ramstrand N.  Prosthetist/orthotist educational experience & professional development in Pakistan.  Disabil Rehabil Assist Technol. 2009 Nov;4(6):385-92. | - To explore areas in which the education at the Pakistan Institute of Prosthetic & Orthotic Science (PIPOS) could be improved or supplemented to facilitate clinical practice of graduates. - To describe educational opportunities PIPOS graduates have had since their graduation and explore their further educational needs. - 15 graduates from PIPOS participated in semi-structured interviews. A qualitative content analysis was applied to the transcripts. | - Respondents indicated a need to upgrade the education at PIPOS. This should include upgrading of resources such as literature and internet access as well as providing staff with the opportunity to further their own education. - Females experienced inequality throughout their education, including in the ability to travel to receive further education. - Limited awareness of the prosthetics and orthotics profession by both the general community and the medical community was also identified as a problem. |
| 109 | Acceptability | - Perceptions of mothers of children with disability regarding rehabilitation | Maloni PK, Despres ER, Habbous J, Primmer AR, Slatten JB, Gibson BE, Landry MD.    Perceptions of disability among mothers of children with disability in Bangladesh: implications for rehabilitation service delivery.  Disabil Rehabil. 2010;32(10):845-54. | - To describe perceptions of disability among mothers of children with disabilities (CWD) in Bangladesh, and to explore how these perceptions influence the care sought for their CWD. - Descriptive qualitative research methods were employed. - Eleven semi-structured interviews were conducted with mothers of CWD receiving services at a large pediatric rehabilitation facility in Bangladesh. - Interviews were recorded and transcribed, and data were coded and analyzed to identify themes. | - Three primary categories of themes emerged: (1) mothers' perceptions of disability; (2) perceptions of treatment; and (3) expectations for the future of their CWD. - The findings suggest that the family members, healthcare providers, and the rehabilitation setting have a considerable influence on mothers' perceptions. - Study participants had adopted a biomedical understanding of disability and treatment, but reported that family elders continued to believe strongly in traditional explanations creating conflict regarding appropriate treatment approaches. - Participants suggested that education and peer support networks provided in the rehabilitation setting played (or could play) a critical role in addressing these conflicts. - Understanding mothers' perceptions of disability and treatment, and the myriad of factors that influence those perceptions, provides valuable knowledge to assist in planning and delivery of family centered rehabilitation services for CWD. |
| 110 | Acceptability | - How local informants can overcome lack of demand in rural communities. | Gona JK, Xiong T, Muhit MA, Newton CR, Hartley S.  Identification of people with disabilities using participatory rural appraisal and key informants: a pragmatic approach with action potential promoting validity and low cost.  Disabil Rehabil. 2010;32(1):79-85. | - The participatory rural appraisal (PRA) and key informant (KI) approaches have been developed and increasingly used in the resource-poor countries. - To investigate the strengths and weaknesses of PRA and KI approaches in the identification of people with disability in resource-poor countries. - A review of published related papers was performed by searching electronic databases including PubMed, Scirus, Health on the Net (HON), Ovid Medline and SOURCE disability database. | - A total of 11 relevant papers were identified from the literature that used PRA or KI methods or both. The PRA and KI approaches were not only consistently less expensive than conventional surveys, but also observed to be simple and fast for identifying disabilities according to local perceptions, although they were less sensitive. - The evidence showed that PRA and KI processes had the benefit of engaging and developing long-term partnerships with the local communities and so the likelihood of positive long-term impact on the community. - The PRA and KI approaches could be fast and cost-effective methods for identifying people with disabilities as an alternative to surveys. They are especially useful when identification is related to subsequent development of community-based services for persons with disabilities. - However, surveys were shown to be more sensitive and therefore more accurate for establishing prevalence rates of impairment. |
| 111 | Quality | - Task-shifting - Advanced care practices | Gillis K, Augruso A, Coe T, O'Neill A, Radford L, Gibson BE, O'Callaghan L, Soever L.  Physiotherapy extended-role practitioner for individuals with hip and knee arthritis: patient perspectives of a rural/urban partnership.  Physiother Can. 2014 Winter;66(1):25-32. | - To explore the perspectives of people with hip and knee arthritis regarding a physiotherapy extended-role practitioner (ERP) model of care in a rural setting. - Using semi-structured interviews, a qualitative descriptive case study was undertaken with 13 participants from a rural family practice located in the province of Ontario, Canada, who had all been assessed by an ERP. - Transcribed interviews were analyzed for emergent themes. | - Three main themes were identified: (1) timely access to care, (2) distance as a factor in seeking care, and (3) perceptions of the ERP model of care. - Participants reported many positive experiences with the physiotherapy ERP rural model. Processes related to minimizing travel required to access care are important for those in rural communities. - Participants reported many positive experiences with the physiotherapy ERP rural model. Processes related to minimizing travel required to access care are important for those in rural communities. |
| 112 | Quality | - Advanced care practices of rehabilitation therapists | Desmeules F, Roy JS, MacDermid JC, Champagne F, Hinse O, Woodhouse LJ.  Advanced practice physiotherapy in patients with musculoskeletal disorders: a systematic review.  BMC Musculoskelet Disord. 2012, Vol. 13, p. 107. | - Evidence of the systematic evaluation of advance physiotherapy practice (APP) models of care is scarce. A systematic review was done to update the evaluation of physiotherapists in APP roles in the management of patients with musculoskeletal disorders. - Structured literature search was conducted in 3 databases (Medline, Cinahl and Embase) for articles published between 1980 and 2011. - Included studies needed to present original quantitative data that addressed the impact or the effect of APP care. A total of 16 studies met all inclusion criteria and were included. | - Advanced care practices can include: communicating a medical diagnosis, triaging patients, ordering of diagnostic tests, conservative treatment recommendations and referral to other health care providers. - Findings are consistent and suggest that APP care may be as (or more) beneficial than usual care by physicians for patients with musculoskeletal disorders, in terms of diagnostic accuracy, treatment effectiveness, use of healthcare resources, economic costs and patient satisfaction. - The emerging evidence suggests that physiotherapists in APP roles provide equal or better usual care in comparison to physicians in terms of diagnostic accuracy, treatment effectiveness, use of healthcare resources, economic costs and patient satisfaction. |
| 113 | Quality | - Extended scope of practice - Advanced care roles | Oakley C, Shacklady C.  The Clinical Effectiveness of the Extended-Scope Physiotherapist Role in Musculoskeletal Triage: A Systematic Review.  Musculoskeletal Care. 2015 Dec;13(4):204-21. | - Extended-scope physiotherapists (ESPs) are working in musculoskeletal (MSK) triage clinics to assess, diagnose and refer patients for appropriate management. However, there is inadequate appraisal of their clinical effectiveness. - The aim of the present systematic review was to appraise the evidence on the diagnostic ability of ESPs in MSK triage, and patient and general practitioner (GP) satisfaction when seen by an ESP in a MSK clinic. - Databases were searched from 1989 to February 2014 using the keywords 'physiotherapy', 'extended practitioner' and 'musculoskeletal disease'. Data extraction was compiled using the Centre for Reviews and Dissemination (2009) method. Diagnostic accuracy studies were assessed for methodological quality using the Scottish Intercollegiate Guideline Network (SIGN) | - From 146 studies initially identified, 14 were eligible for review. Only one diagnostic study was of high quality, and satisfaction study scores ranged from 40% to 73%. - All studies reported favourable outcomes for ESPs in MSK triage clinics, with ESPs demonstrating a good level of diagnostic ability in comparison with a gold standard such as surgery. - In addition, patients and GPs were satisfied with the overall performance and service provided by ESPs. - The evidence suggests that ESPs are clinically effective. |
| 114 | Quality | - Advanced care practices - Military | Rhon D, Deyle GD, Gill NW.  Clinical reasoning and advanced practice privileges enable physical therapist point-of-care decisions in the military health care system: 3 clinical cases.  Phys Ther. 2013, Vol. 93(9), pp. 1234-43 | - Physical therapists frequently make important point-of-care decisions for musculoskeletal injuries and conditions. In the Military Health System (MHS), these decisions may occur while therapists are deployed in support of combat troops, as well as in a more traditional hospital setting. Proficiency with the musculoskeletal examination, including a fundamental understanding of the diagnostic role of musculoskeletal imaging, is an important competency for physical therapists. - To present 3 cases managed by physical therapists in unique MHS settings | - Three cases are presented involving conditions where the physical therapist was significantly involved in the diagnosis and clinical management plan. - The physical therapist's clinical privileges, including the ability to order appropriate musculoskeletal imaging procedures, were helpful in making clinical decisions that facilitate timely management. - The cases involve patients with an ankle sprain and Maisonneuve fracture, a radial head fracture, and a pelvic neoplasm referred through medical channels as knee pain. - Physical therapists can provide important contributions to the primary management of patients with musculoskeletal conditions in a variety of settings within the MHS. In the cases described, advanced clinical privileges contributed to the success in this role. |
| 115 | Quality | - Credentials - Qualifications - Clinical doctorate - Entry-to-practice - Occupational Therapy | Brown T, Crabtree JL, Mu K, Wells J.  The entry-level occupational therapy clinical doctorate: advantages, challenges, and international issues to consider.  Occup Ther Health Care. 2015 Apr;29(2):240-51 | - Perspective paper on the challenges to consider about the entry-level professional credential to practice, in case with a focus on the occupational therapy clinical doctorate. - The article reviews current literature and discusses issues about the occupational therapy entry-level clinical doctorate. - Summarizes frequently cited pros and cons of moving to the clinical doctorate as the singular entry point to occupational therapy practice are considered. | - It outlines the recommendation under consideration by the American Occupational Therapy Association (AOTA) that by 2025, all occupational therapy university programs will move to the clinical doctorate level. - Meanwhile, different levels of university programs quality for OT professional practice. |
| 116 | Quality | - Task Shifting - Therapist assistants - Advanced care activities - Lower-level care activities | Hsieh CH, Putman K, Nichols D, McGinty ME, DeJong G, Smout RJ, Horn S.  Physical and occupational therapy in inpatient stroke rehabilitation: the contribution of therapy extenders.  Am J Phys Med Rehabil. 2010 Nov;89(11):887-98. | - To understand the use of therapy extenders in stroke rehabilitation. - Descriptive analysis of a prospective observational cohort study. | - Physical and occupational therapists spend more time in delivering advanced activities that include ongoing integrated evaluation and treatment planning or modification. - Their assistants spend more time in delivering lower-level activities, such as bed mobility, transfers, dressing, or nonfunctional activities. - Also, therapists are more likely to assign responsibility to assistants to treat moderate motor impairment among patients with stroke. |
| 117 | Quality | - Task shifting - New allied health assistant role - Practice patterns | Stute M, Hurwood A, Hulcombe J, Kuipers P.  Pilot implementation of allied health assistant roles within publicly funded health services in Queensland, Australia: results of a workplace audit.  BMC Health Serv Res. 2014, Vol. 14, p. 258. | - Allied health assistants provide delegated support for physical therapists, occupational therapists and other allied health professionals. Unfortunately the role statements, scope of practice and career pathways of these assistant positions are often unclear. - To inform the future development of the allied health assistant workforce, a state-wide pilot project was implemented and audited. - New allied health assistant positions were implemented in numerous settings at three levels (trainee level, full (standard) scope and advanced scope level). Six months after implementation, 41 positions were audited, using a detailed on-site audit process, conducted by multiple audit teams. | - Both the full (standard) scope and the advanced scope positions were warranted, however the skills of the allied health assistants were not optimally utilised. - Contributing factors to this underutilization included the reluctance of professionals to delegate clinical tasks, inconsistencies in role descriptions, limitations in training, and the time frame taken to reach an effective skill level. - Optimal utilisation of assistants is unlikely to occur while professionals withhold delegation of tasks related to direct patient care. - Formal clinical supervision arrangements and training plans should be established in order to address the concerns of professionals and accelerate full utilisation of assistants. - Further work is necessary to identify the key components and distinguish key features of an advanced allied health assistant role. |
| 118 | Quality | - Home health Aids - Task-Shifting - Expanded roles: supported | Guay M, Dubois MF, Desrosiers J.  Can home health aids using the clinical algorithm Algo choose the right bath seat for clients having a straightforward problem?  Clin Rehabil. 2014 Feb;28(2):172-82. | - To determine if Algo, a clinical algorithm to select bathing equipment for 'straightforward' cases, guides home health aides in selecting the appropriate bath seat. - Criterion validity study in a community home care setting. - Eight home health aides used Algo with community-dwelling older adults were the subjects. Their bath-seat recommendations were compared with those proposed by an occupational therapist (OT), which were considered as the gold standard. Another OT was also used. | - In 84% of cases (95% confidence interval (CI) = [75, 93]), the non-OTs using Algo identified a seat that would enable these older adults to bathe according to their preferences, abilities and environment, as confirmed by the gold standard OT. - Moreover, this appropriateness rate did not statistically differ from that obtained when comparing another OT to the gold standard. |
| 119 | Quality | - Occupational Therapy Assistants - Task Shifting - Expanded support worker: roles | Cox RJ, Mills VJ, Fleming J, Nalder E.  Implementation of an advanced occupational therapy assistant-led groups programme in aged care rehabilitation.  Aust Occup Ther J. 2014 Jun;61(3):187-93 | - To implement an advanced occupational therapy assistant-led groups programme in a subacute aged care rehabilitation setting and to evaluate the impact on the clinical outcomes - Prospective quasi-experimental cohort study comparing outcomes of 30 patients receiving a groups programme led by an advanced occupational therapy assistant with a historical control group of 40 patients receiving the groups programme led by an occupational therapist. | - Outcomes of patients participating in the assistant-led groups programme were not significantly different to patients who participated in the therapist-led groups programme. - The introduction of an advanced occupational therapy assistant to replace an occupational therapist in facilitating a groups programme in aged care rehabilitation did not result in a decline in patient outcomes. |
| 120 | Quality | - Task shifting - Supervision of assistants | Resnik L, Feng Z, Hart DL.  State regulation and the delivery of physical therapy services.  Health Serv Res. 2006, Vol. 41(4 Pt 1), pp. 1296-316. | - To examine the relationship between state regulations of physical therapists (PT) and three dependent variables: physical therapist assistant (PTA) utilization more than 50 percent of the time during the treatment episode (high PTA utilization), number of visits, and patient self-reported functional health status (FHS) at discharge. - The analytic sample included 63,900 patients from 38 states drawn from 395 clinics who participated in the Focus on Therapeutic Outcomes Inc. (Knoxville, TN) database in 2000 and 2001 - Using a Bayesian modeling approach with the Markov Chain Monte Carlo estimation method, we fitted separate multilevel multivariate regression models predicting high PTA utilization, number of visits, and discharge FHS. | - After controlling for patient, therapist, and clinic characteristics, the presence of state regulations regarding PTA supervision was not associated with the likelihood of high PTA utilization. - High PTA utilization and regulations requiring full-time onsite supervision were associated with more visits, whereas regulation of PT/PTA ratio was associated with fewer visits. - Supervisory regulations were associated with better discharge FHS. High PTA utilization and use of therapy aides were associated with more visits per episode and lower discharge FHS. - The use of care extenders in place of PTs is likely to result in less efficient and lower quality care in outpatient rehabilitation. |
